# Using artificial intelligence to assess personal qualities in college admissions

**DOI:** 10.1126/sciadv.adg9405

**Published:** 2023-10-12

**Authors:** Benjamin Lira, Margo Gardner, Abigail Quirk, Cathlyn Stone, Arjun Rao, Lyle Ungar, Stephen Hutt, Louis Hickman, Sidney K. D’Mello, Angela L. Duckworth

**Affiliations:** ^1^ University of Pennsylvania, Philadelphia, PA, USA.; ^2^ University of Colorado-Boulder, Boulder, CO, USA.; ^3^ University of Denver, Denver, CO, USA.; ^4^ Virginia Tech, Blacksburg, CO, USA.

## Abstract

Personal qualities like prosocial purpose and leadership predict important life outcomes, including college success. Unfortunately, the holistic assessment of personal qualities in college admissions is opaque and resource intensive. Can artificial intelligence (AI) advance the goals of holistic admissions? While cost-effective, AI has been criticized as a “black box” that may inadvertently penalize already disadvantaged subgroups when used in high-stakes settings. Here, we consider an AI approach to assessing personal qualities that aims to overcome these limitations. Research assistants and admissions officers first identified the presence/absence of seven personal qualities in *n* = 3131 applicant essays describing extracurricular and work experiences. Next, we fine-tuned pretrained language models with these ratings, which successfully reproduced human codes across demographic subgroups. Last, in a national sample (*N* = 309,594), computer-generated scores collectively demonstrated incremental validity for predicting 6-year college graduation. We discuss challenges and opportunities of AI for assessing personal qualities.

## Teaser

Artificial intelligence can be used to measure personal qualities in college admissions equitably and transparently.

## INTRODUCTION

Many colleges embrace the ideals of holistic review. In a recent survey by the National Association for College Admissions Counseling, 70% of admissions officers said that they consider personal qualities to be an important factor when selecting applicants (*1*). This aim is justified by longitudinal research affirming that personal qualities, whether referred to as “noncognitive skills,” “social-emotional competencies,” “personality,” or “character,” predict positive life outcomes in general and success in college in particular (*2*–*5*). Moreover, a holistic admissions process can advance equity, some argue, as applicants are able to demonstrate qualifications not reflected in their standardized test scores, which tend to be highly correlated with socioeconomic advantage (*6*).

However, history shows that equity is certainly not guaranteed by holistic review. A century ago, the Columbia University first began requiring applicants to write a personal essay, which admissions officers evaluated for evidence of “good character” (*7*). Previously, the university’s admissions decisions had been based primarily on standardized test scores. The result was a growing proportion of Jewish students in each entering class, which in turn led to concerns that, as Columbia’s dean at the time put it, the campus was no longer welcoming to “students who come from homes of refinement” (p. 87). It has been argued that for Columbia and other Ivy League colleges in that era, not requiring the justification, explanation, or even disclosure of these summary character judgments enabled the unfair exclusion of qualified Jewish applicants.

Although its aims may be nobler today, the holistic review process itself remains much the same. admissions officers still rely heavily on the personal essay to evaluate an applicant’s personal qualities (*1*). The particulars of how, or even which, personal qualities are assessed, remain undisclosed to either applicants or the public, and even the “admissions officers themselves simply do not have a common definition of holistic review beyond ‘reading the entire file’” (*8*). As one admissions officer put it, the status quo of holistic review is both “opaque and secretive” (*9*).

Recently, a more transparent and systematic process has been recommended for the holistic review of personal qualities in college admissions. Specifically, admissions officers have been urged to assess individual personal qualities separately (as opposed to making a summary judgment of good character), to use structured rubrics (as opposed to intuition), and to carry out multiple, independent evaluations (as opposed to relying on a single officer’s judgment) (*6*, *10*). Such recommendations represent the application of basic psychometric principles and, in research contexts, have long been used to increase the reliability, validity, and interpretability of human ratings (*11*, *12*). Moreover, the transparency of this systematic approach should limit bias, whether accidental or intentional.

In college admissions, however, this ideal is hardly ever achieved. The soaring number of applications that admissions officers must review, which for the majority of colleges has more than doubled in the last two decades, affords extraordinarily limited time to review each one (*13*, *14*). These logistical and budgetary constraints are likely to continue to prohibit the implementation of best practices that, were resources unlimited, could optimize reliability, validity, interpretability, and in turn, equity.

Can artificial intelligence (AI) advance the aims of holistic review? With stunning efficiency, AI systems identify patterns in data and, with stunning fidelity, apply learned models to new cases. For example, a computer algorithm could be trained to generate personal quality scores from student writing instantaneously, reliably, and at near-zero marginal cost. However, there are concerns that the “black box” of an AI algorithm may inadvertently perpetuate, or even exacerbate, bias against disadvantaged subgroups (*15*, *16*). Such bias has been shown in the domains of hiring, criminal justice, and medical diagnosis (*17*–*19*). In college admissions, AI-quantified essay content and style have been shown to correlate more strongly with household income than do SAT scores (*20*). Opaque AI algorithms that provide fertile ground for bias recall the anti-Semitic holistic review practices of a century ago.

Efforts within the AI community to address these issues have given rise to concepts such as human-centered AI (*21*, *22*) and explainable AI (*23*). These frameworks emphasize alignment with stakeholder objectives, interpretability, and equity, while promoting the idea of automation as a complement rather than a substitute for human control (*22*). Rather than simply maximizing predictive accuracy, these approaches prioritize alignment with stakeholder goals [e.g., admitting students who demonstrate prosocial purpose (*24*)], interpretability (e.g., providing separate, face-valid scores for separate personal qualities rather than a single summary score of character with no evidence of face validity), and rigorously auditing model outputs for unintended bias. By prioritizing these aspects, digital technology can facilitate the identification of discrimination and contribute to rectifying historical exclusion (*25*).

**Fig. 1.**
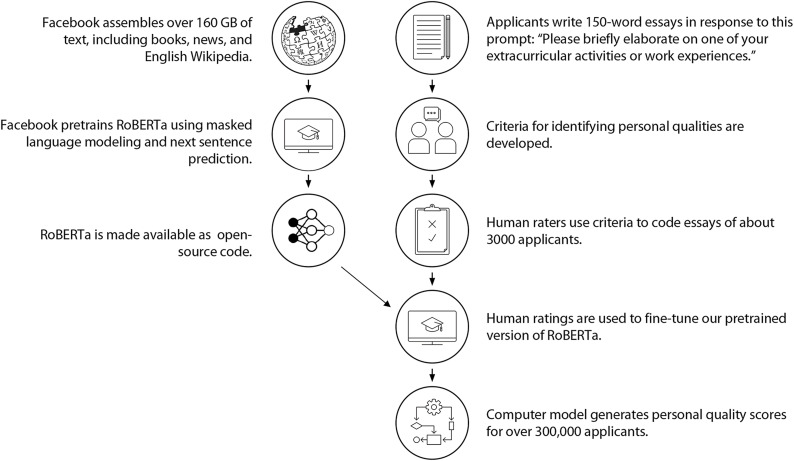
An AI approach to assessing personal qualities in college admissions.

In this investigation, we developed an AI approach to assessing personal qualities with these priorities in mind. We began with a de-identified sample of 309,594 college applications (see Fig. 1). Each included a 150-word essay describing an extracurricular or work activity of the applicant’s choice. Next, in a development sample of 3131 essays, research assistants and admissions officers identified the presence or absence of seven different personal qualities commonly valued by universities and shown in prior research to predict college success (*3*). See Table 1. Research assistant and admissions officer ratings were used to fine-tune separate Robustly Optimized BERT Pretraining Approach (RoBERTa) language models (*26*) for each personal quality. We then confirmed each model’s interpretability and evidence of convergent, discriminant, and predictive validity by demographic subgroup. Last, we applied these fine-tuned models to the holdout sample of 306,463 essays, examining associations between computer-generated personal quality scores, demographic characteristics, and 6-year college graduation.

**Table 1.** Personal qualities and example essay excerpts. Note: Our data use agreement with Common App does not allow us to publish real excerpts to protect student identity.

Personal quality	Fictionalized excerpts
**Prosocial purpose** Helping others, wanting to help others, considering the benefits to others, mentioning reasons for helping others, or reflecting on how enjoyable or rewarding it is to help others.	Every summer for the last 3 years, I worked as camp counselor at a camp for young children from underprivileged families. Helping children realize their hidden talents is one of the most rewarding experiences I have ever had. I’ve been so fulfilled by watching these children develop confidence in their abilities. This experience has been so important to me, and it showed me that a career in education is where I belong.
**Leadership** Serving in a leadership role, commenting on what he or she did in his or her capacity as a leader, or discussing the value, meaning, or importance of leadership.	I was chosen to be cheerleading captain during my senior year. My freshman year captain had a huge impact on my life, and I felt like it was my time to pay it forward. I am so proud of everything I did for the girls: creating a mentorship system, organizing events and fundraisers, and encouraging everyone to work as hard as they could. At the end of the year, a few girls thanked me. I was completely overcome with emotion. I’ve never felt so gratified in my life.
**Learning** Improving, learning, or developing knowledge, skills, or abilities.	I played softball in high school. When I started, I was not a very strong player. When I finally made the varsity team my senior year, I was determined to have a better season. I worked constantly to improve my game - during practice and on my own time. My skills grew so much. Because of my hard work, I finished the year with the best record on my team!
**Goal pursuit** Having a goal and/or a plan.	I have been playing soccer since I was 6 years old. Unfortunately, last year I injured my knee, and it has been a struggle to get back to the level I was playing at before my injury. It has been really challenging, but I’ve been doing physical therapy and practicing everyday so that I can be a varsity starter this year.
**Intrinsic motivation** Describing the activity as enjoyable or interesting. Liking the activity or identifying with it.	Running track is so much more than a sport to me. It’s a challenge and an adventure, and I put everything I have into it. I love every aspect of it, even the afternoons I spend drenched in sweat in the scorching heat.
**Teamwork** Working with or learning from others. Valuing what fellow participants bring to the activity.	I’ve been on my school’s debate team since my freshman year, and was elected co-captain because of my commitment to the team’s success. My fellow co-captains and I worked together to get our team ready for competitions. We knew that a strong team performance was more important than the successes of a few individuals. We stressed teamwork and cooperation between our teammates. Because we focused on team effort, we earned first place at the state meet.
**Perseverance** Persisting in the face of challenge.	I’ve learned to become a gracious victor and to grow from defeat. Track has helped me overcome my fear of losing, and even helped me put my life in perspective. I’ve learned to keep working and fighting even when the odds seem impossible to beat. There were many times that I found myself lagging, but I pulled ahead at the end because I never gave up. The most important thing I’ve learned is to never let anything stand in my way.

## RESULTS

On average, research assistants and admissions officers found evidence for two of seven personal qualities in each essay. As shown in Table 2, some personal qualities were more commonly observed than others. For instance, research assistants and admissions officers identified leadership in 42 and 44% of essays, respectively; in contrast, they identified perseverance in only 19 and 21% of essays, respectively. Correlations between research assistant and admission officer ratings ranged between ϕ = 0.193 and 0.703, *P*s < 0.001.

**Table 2.** Descriptive statistics and correlations between human ratings and computer-generated likelihoods of personal qualities in the development sample. Note: Inter-rater reliability for human raters was measured with Krippendorf’s α. Correlations between human ratings and computer-generated likelihoods for the same personal qualities are shown along the diagonals. All correlations are point-biserial correlation coefficients between binary human ratings and continuous computer-generated likelihoods. *n* = 3131. *n* for inter-rater reliability = 206 essays coded by multiple research assistants, and *n* = 3131 essays coded by two admission officers. PP, prosocial purpose; LD, leadership; TW, teamwork; LR, learning; PS, perseverance; IM, intrinsic motivation; GP, goal pursuit.

Personal quality	Research assistant ratings							Admissions officer ratings						
	PP	LD	TW	LR	PS	IM	GP	PP	LD	TW	LR	PS	IM	GP
Computer-generated likelihoods														
1. PP	0.86***	−0.01	−0.04*	−0.09***	−0.12***	−0.05**	0.04*	0.80***	−0.13***	0.13***	−0.22***	−0.24***	−0.09**	−0.19***
2. LD	−0.01	0.81***	0.15***	−0.01	0.00	−0.09***	0.05**	0.13***	0.73***	0.16***	−0.15***	−0.06***	−0.16***	0.01
3. TW	−0.07***	0.18***	0.62***	0.06**	0.07***	−0.02	0.06**	−0.18***	0.16***	0.62***	0.07***	0.10***	−0.03	0.10***
4. LR	−0.10***	−0.05**	0.04*	0.77***	0.11***	−0.01	−0.03	−0.28***	−0.15***	−0.07***	0.65***	0.07***	0.01	0.09***
5. PS	−0.16***	−0.01	0.06**	0.10***	0.67***	0.03	0.05**	−0.35***	−0.10***	0.11***	0.08***	0.48***	0.09***	0.26***
6. IM	−0.05**	−0.09***	0.00	−0.03	0.04*	0.73***	0.03	0.08*****	−0.24***	−0.08***	−0.01	0.08***	0.45***	−0.05***
7. GP	0.06***	0.06**	0.06***	−0.01	0.02	0.02	0.59***	−0.31***	−0.05**	0.12***	0.15***	0.27***	0.06***	0.45***
Descriptive statistics														
Human inter-rater reliability	0.83	0.78	0.61	0.73	0.66	0.63	0.57	0.60	0.49	0.30	0.31	0.24	0.23	0.15
Frequency of human rating	0.34	0.18	0.26	0.42	0.19	0.42	0.31	0.28	0.25	0.22	0.44	0.21	0.41	0.25
Mean of computer-generated likelihood	0.36	0.19	0.26	0.45	0.19	0.45	0.32	0.30	0.25	0.22	0.46	0.24	0.42	0.25

*P < 0.05.

**P < 0.01.

***P < 0.001.

Using these binary human ratings, we fine-tuned separate RoBERTa models to produce continuous likelihood scores for each personal quality and each kind of rater. See section S2 in for details on model pretraining and fine-tuning.

### Model interpretability

**Fig. 2.**
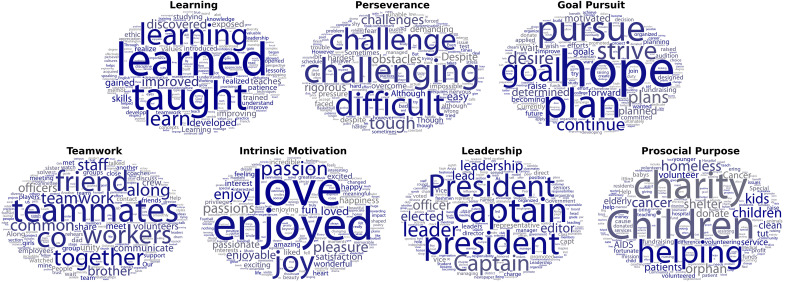
Complete or partial words on which RoBERTa models fine-tuned on research assistants relied most for generating personal quality scores. Font size is proportional to word importance. Darker words are more common. Token “gru” is a fraction of the word “grueling,” and token “unte” is a fraction of the word “volunteer.” Words importance is not invariant across essays, it depends on word context. Word importance and frequency were largely independent (*r* = −0.03 and *P* < 0.001). For instance, for intrinsic motivation, the model relied more on the word “pleasure” then the word “fun,” but essays were more likely to contain the word “fun” then the word “pleasure.”

We used the transformers-interpret package (*27*, *28*) to identify the words (or fractions of words) that these fine-tuned RoBERTa models relied on most to generate personal quality scores. As shown in Fig. 2, there was reasonable evidence of face validity. For instance, RoBERTa assigned higher scores for leadership when essays mentioned “president,” “leader,” and “captain.” Models trained on admission officer ratings produced similar attribution scores: average word-level attribution scores correlated between 0.392 and 0.983, *P*s < 0.001. See section S7 for details.

### Convergent and discriminant validity of computer-generated likelihoods in the development sample

Computer-generated likelihoods for each personal quality converged with human ratings of the same personal quality (*r*s ranged from 0.59 to 0.86, average *r* = 0.74 for research assistants; *r*s ranged from 0.45 to 0.80, average *r* = 0.62 for admission officers). In contrast, computer-generated likelihoods for a particular personal quality did not correlate with human ratings of other personal qualities (*r*s from −0.16 to 0.18, average *r* = 0.01 for research assistants; *r*s from −0.35 to 0.27, average *r* = −0.03 for admission officers). See Table 2. As expected, the more reliably human raters were able to code each personal quality, the better the computer-generated likelihoods of personal qualities matched these ratings (*r* = 0.95 and *P* = 0.001 for research assistants; *r* = 0.94 and *P* = 0.001 for admission officers). In the subsample of essays that were coded by multiple raters, model scores correlated more strongly with human ratings than human ratings correlated with each other (*M*_human−computer_ = 0.74, *M*_human−human_ = 0.69, *t* = 4.16, and *P* = 0.006 for research assistants; *M*_human−computer_ = 0.60, *M*_human−human_ = 0.28, *t* = 19.40, and *P* < 0.001 for admissions officers). There were positive correlations between computer-generated likelihoods for personal qualities from models trained on research assistants and admissions officers (*r*s ranged from 0.394 to 0.869, *P*s < 0.001).

### Convergent validity does not vary by demographic subgroup in the development sample

Correlations between human ratings and computer-generated likelihoods of personal qualities were similar across subgroups. For example, the average correlation between human-rated and computer-generated personal quality scores was 0.74 for female applicants and 0.73 for male applicants for research assistants. The pattern of results was equivalent for admission officers. As shown in tables S11 and S12, after correcting for multiple comparisons (*29*), 13 and 9% of the correlations differed by subgroup for research assistants (RAs) and admissions officers (AOs), respectively. In about half of these comparisons, the models were more accurate for the marginalized group, while in the other half, the majority subgroup was favored. In most of these cases, the difference between the correlations was not very large (mean ∣Δ*r*_RA_∣ = 0.054 and range of Δ*r*_RA_ = −0.121 to 0.056); and mean ∣Δ*r*_AO_∣ = 0.10 and range of Δ*r*_AO_ = −0.206 to 0.123).

### Human ratings and computer-generated likelihoods were largely unrelated to demographics in the development sample

Demographic characteristics were largely unrelated to personal qualities, whether assessed by human raters (mean |ϕ_RA_| = 0.02 and mean |ϕ_AO_| = 0.03) or by computer algorithm (mean |*d*_RA_| = 0.06 and mean |*d*_AO_| = 0.08). One exception is that female applicants were rated as more prosocial than male applicants [ϕ_RA_ = 0.13, ϕ_AO_ = 0.12, and *P* < 0.001 for human ratings, *d*_RA_ = 0.26, *d*_AO_ = 0.28, and *P* < 0.001 for computer-generated likelihoods, *P* values adjusted for multiple comparisons (*29*)], in line with other research showing gender differences in prosocial motivation and behavior favoring women (*30*). See table S5 for details.

### Computer-generated likelihoods were as predictive of college graduation as human raters in the development sample

To compare the predictive validity of the computer-generated likelihoods with human ratings, we ran two logistic regression models in which personal qualities predicted college graduation. The computer-generated likelihoods were slightly more predictive than the human ratings, but the difference in the areas under the curve (AUCs) was not significant (AUC_human_ = 0.565, AUC_computer_ = 0.574, ΔAUC = 0.009, and *P* = 0.274 for research assistants; AUC_human_ = 0.587, AUC_computer_ = 0.603, ΔAUC = 0.017, and *P* = 0.120 for admission officers). Coefficients were slightly larger for computer-generated likelihoods as compared to human ratings (*t* = 2.33, *d* = 0.882, and *P* = 0.059 for research assistants; *t* = 3.89, *d* = 1.469, and *P* = 0.008 for admissions officers).

### Computer-generated likelihoods were largely independent of demographics but, in support of criterion validity, predicted graduation in the holdout sample

Next, we applied the models fine-tuned on research assistants and admissions officers to the holdout sample of 306,463 essays. For both categories of models, reliability across models trained on different subsets of the data was high (range of Cronbach’s α = 0.990 to 0.997 for research assistants; range of Cronbach’s α = 0.988 to 0.998 for admission officers). Even when considering any two models, they were likely to produce similar results (average intermodel correlation ranged from 0.910 to 0.967 for research assistants and 0.896 to 0.978 for admission officers). Correlations between computer-generated likelihoods for personal qualities from models trained on research assistants and admissions officers ranged from 0.418 to 0.896, *P*s < 0.001.

As in the development sample, computer-generated likelihoods for personal qualities were similar across demographic subgroups (mean |*d*_RA_| = 0.05 and mean |*d*_AO_| = 0.06). In contrast, and as expected, demographics were more strongly related to standardized test scores (mean |*d*| = 0.38) and degree of participation in out-of-school activities (mean |*d*| = 0.17). See tables S7 and S10 for details.

About 78% of students in the holdout sample graduated from college within 6 years. As shown in model 1 in Table 3, computer-generated likelihoods for personal qualities were each modestly predictive of college graduation when controlling for each other [odds ratios (ORs) from 1.041 to 1.132, *P*s < 0.001, and AUC = 0.560 for research assistants; ORs from 1.048 to 1.252, *P*s < 0.001, and AUC = 0.576 for admission officers]. To estimate a ceiling on how much the essays can predict subsequent college graduation, we trained a RoBERTa model to predict college graduation from students’ responses. This model achieved an out-of-sample AUC of 0.626, suggesting that consistent with previous research (*20*) essays do encode information predictive of graduation outside of personal qualities. The same procedure using personal qualities results in smaller out-of-sample AUCs (AUC_RA_ = 0.557 and AUC_AO_ = 0.568). See section S8 for details.

**Table 3.** ORs from binary logistic regression models predicting 6-year college graduation in the *N* = 306,463 holdout sample.

	Research assistant		Admission officer	
	(1)	(2)	(1)	(2)
Computer-generated likelihoods of personal qualities				
Prosocial purpose	1.132***	1.075***	1.252***	1.116***
	(0.005)	(0.005)	(0.006)	(0.006)
Leadership	1.133***	1.065***	1.214***	1.084***
	(0.005)	(0.005)	(0.005)	(0.005)
Teamwork	1.080***	1.031***	1.135***	1.062***
	(0.005)	(0.005)	(0.005)	(0.005)
Learning	1.065***	1.045***	1.146***	1.034***
	(0.004)	(0.005)	(0.005)	(0.005)
Perseverance	1.071***	1.012**	1.089***	1.047***
	(0.005)	(0.005)	(0.005)	(0.006)
Intrinsic motivation	1.068***	1.007	1.142***	1.009
	(0.004)	(0.005)	(0.005)	(0.005)
Goal pursuit	1.041***	1.005	1.048***	1.030***
	(0.004)	(0.005)	(0.005)	(0.005)
Race/ethnicity (vs. white)				
Black		0.774***		0.775***
		(0.019)		(0.019)
Latino		0.871***		0.868***
		(0.019)		(0.019)
Asian		0.735***		0.739***
		(0.017)		(0.017)
Other		0.749***		0.750***
		(0.017)		(0.017)
No race reported		0.849***		0.853***
		(0.013)		(0.013)
Parental education (vs. no parent w/ college degree)				
One parent w/ college degree		1.199***		1.198***
		(0.012)		(0.012)
Two parents w/ college degree		1.335***		1.334***
		(0.012)		(0.012)
Female		1.435***		1.430***
		(0.010)		(0.010)
Married parents		1.311***		1.308***
		(0.011)		(0.011)
English language learner		0.769***		0.774***
		(0.015)		(0.016)
Title 1 high school		0.951***		0.947***
		(0.013)		(0.013)
Out-of-school activities (OSA)				
Number of OSA		1.250***		1.241***
		(0.005)		(0.005)
Time per OSA		1.088***		1.083***
		(0.004)		(0.004)
Proportion sports		1.042***		1.035***
		(0.005)		(0.005)
Standardized test scores		1.489***		1.482***
		(0.006)		(0.006)
Constant	3.555***	2.533***	3.585***	2.543***
	(0.004)	(0.014)	(0.004)	(0.014)
AUC	0.560	0.689	0.576	0.690

*P < 0.05.

**P < 0.01.

***P < 0.001.

As shown in model 2 in table S4, in the models trained on research assistants, five of seven personal qualities remained predictive of college graduation when controlling for each other, demographics, standardized test scores, and out-of-school activities (ORs from 1.012 to 1.075 and *P*s < 0.01). In the models trained on admissions officers, six of seven personal qualities remained predictive (ORs from 1.030 to 1.116 and *P*s < 0.01). See fig. S2 for details on imputation.

As a further test for fairness, we tested whether the predictive power of computer-generated likelihoods of personal qualities was equivalent across subgroups. We added interaction terms between each personal quality and standardized test scores and each demographic characteristic. After controlling for multiple comparisons (*29*), we confirmed that the predictive effect of personal qualities was equal across demographic subgroups. Comparatively, the predictive accuracy of standardized tests differed across subgroups (mean ∣β∣ = −0.053). We also tested for differences in predictive validity in intersections of two demographic subgroups (e.g., Black English language learners and women in title 1 high schools). There were no consistent or theoretically interpretable patterns in these intersectional analyses. See section S9 for details.

## DISCUSSION

In a national dataset of over 300,000 college applications, we evaluated an AI approach to measuring personal qualities from student writing. Specifically, we fine-tuned RoBERTa language models using expert ratings of prosocial purpose, leadership, teamwork, learning, perseverance, intrinsic motivation, and goal pursuit, respectively, in applicants’ essays about their out-of-school activities. We found that these models demonstrated convergent, discriminant, and predictive validity, and this evidence was consistent across demographic subgroups. In addition, computer-generated scores were largely independent of demographics.

In contrast, two prior studies found that AI-extracted admission essay content and style correlate with socioeconomic status. Alvero *et al.* (*20*) found that students from wealthier families tend to write about certain essay topics (e.g., human nature), whereas disadvantaged students tend to write about others (e.g., tutoring groups). Likewise, Pennebaker *et al.* (*31*) found that categorical words (e.g., articles, prepositions) versus dynamic words (e.g., pronouns, adverbs) in college essays correlate with parental education at *r* = 0.22. Why do our results differ? It seems likely that personal qualities are distributed more evenly across demographic subgroups than the topics students choose to write about or the words they use to do so. However, we cannot rule out methodological differences. Alvero *et al.* (*20*) used essays from the University of California system, and Pennebaker *et al.* (*31*) used essays from a large state university. In contrast, our sample included a larger and more diverse set of public and private 4-year colleges from across the United States. In addition, both of these prior studies used personal statements totaling several hundred words, whereas the essays to which we had access were a maximum of 150 words and focused specifically on extracurricular activities and work experiences. Last, rather than using unsupervised topic modeling or dictionary approaches, we fine-tuned a language representation model using human ratings that themselves were shown to be unbiased.

Several limitations of this investigation suggest promising directions for future research. First, while our national dataset was unusually large and diverse, it did not include the 650-word personal essay now required by the Common Application. Unfortunately, applicants in 2008 to 2009 submitted their personal essays as attached PDF files that were not feasible to de-identify. A replication and extension of our study using a more recent cohort of applicants should not face this limitation.

Second, and relatedly, because the majority of applicants in our sample submitted their high school transcripts as attached PDF files that could not be de-identified, our dataset included high school GPAs for only a subsample of 43,592 applicants whose school counselors entered grades directly into the Common Application online portal. While our robustness check using this subsample (see table S52) affirms the conclusions of our main analyses, future research should not face this limitation.

Third, the observed effect sizes for personal qualities predicting college graduation were modest, both in absolute terms and relative to the predictive validity of standardized test scores. They were, however, somewhat larger than predictive validities of questionnaire measures of personal qualities like growth mindset (*32*). As context, a growing literature suggests that long-term life outcomes are extremely difficult to predict with precision (*33*, *34*), in part because the greater the number of factors that determine an outcome, the smaller the influence of any single one (*35*, *36*). Relatedly, it is worth noting that myriad factors unmeasured in this investigation have been shown to influence college graduation, including the ability to afford tuition payments (*37*), academic preparation and support (*38*, *39*), and sense of belonging (*32*, *40*).

Fourth, college graduation was the only outcome available in our dataset. We therefore could not evaluate the impact of personal qualities on other aspects of college success, such as GPA, extracurricular involvement, and contributions to the campus community, nor on social or emotional well-being (*41*). This limitation, while not atypical, illuminates a more general concern with research on college admissions, namely, the lack of explicit, consensual priorities for what college admissions decisions are aimed at optimizing and how such outcomes are operationalized.

One unexpected benefit of evaluating AI approaches, therefore, is the critical perspective brought to the current status of holistic review and selective admissions. Thus, future research and practice should focus on clarifying the goals of holistic review (*8*) before automating parts of the process.

Last, inter-rater reliability estimates and human-computer correlations were lower for admissions officers than for research assistants. These disparities may reflect differences in methodology (e.g., research assistants received more training on the coding instructions) or in rater perspective (e.g., heterogeneity in admission officers’ ratings may reflect differences in the priorities of their universities). Our data do not distinguish between these possibilities. Regardless, it seems likely that the more reliable ratings of research assistants provided a more consistent signal for the models to learn from, resulting in higher human-computer correlations for research assistants compared to admissions officers. Notably, computer-generated scores for personal qualities were at least as, if not more, predictive of college graduation when the algorithm was trained by admissions officers as when it was trained by research assistants. While unexpected, this pattern of results underscores the fact that increasing reliability does not always increase validity. By analogy, a questionnaire can achieve nearly perfect internal reliability when items are practically synonymous but only at the cost of content and predictive validity (*42*).

In summary, this investigation suggests that an AI approach to measuring personal qualities warrants both optimism and caution. On one hand, our findings demonstrate that AI models trained on human ratings are not only efficient (yielding millions of personal quality scores in a matter of minutes, replicating human ratings with uncanny precision) but also interpretable (as opposed to an inscrutable black box) and auditable for fairness to demographic subgroups. On the other hand, Campbell’s law (*43*) states that the more weight given to an assessment in high-stakes decisions (as opposed to low-stakes research), the greater the incentive for distortion. It is not hard to imagine how applicants might try to mold their essays, perhaps using AI tools such as ChatGPT, to match what admissions officers, and the algorithms they train, are looking for. We can only assume that applicants from more advantaged backgrounds would be better positioned to do so. What is more, algorithms make mistakes, in particular insofar as they look for patterns and thus, by design, are blind to exceptions. For instance, our fine-tuned RoBERTa model gives the sentence “I donated heroin to the children’s shelter” an extremely high score for prosocial purpose. Thus, we recommend AI be used to augment, not replace, human judgment. No algorithm can decide what the goals of a university’s admissions process should be or what personal qualities matter most for that community. Seeing algorithms as complements rather than replacements for human judgment may also counter algorithm aversion, the tendency to trust human decision-makers over algorithms, even in the face of contradictory evidence (*44*). With these caveats in mind, we conclude with the observation that progress in any field depends on dissatisfaction with the status quo; there is no doubt that when it comes to the assessment of personal qualities in college admissions, we can do better.

## MATERIALS AND METHODS

### Participants

After exclusions, our sample consisted of 309,594 students who applied to universities in 2008 to 2009. To provide labeled data for the machine learning algorithm, we set aside a development sample consisting of 3131 applications for manual coding. We used stratified random sampling to ensure representation across demographic groups and levels of involvement in extracurricular activities. The holdout sample was composed of the remaining 306,463 essays. We applied the fine-tuned algorithm to these essays and tested the relationship between the computer-generated likelihoods of personal qualities and demographics as well as college graduation. See section S1 for details on missing data and exclusion criteria.

### Measures

#### Extracurriculars essay

In up to 150 words, applicants who completed the Common Application were asked to respond to the following prompt: “Please briefly elaborate on one of your activities or work experiences.” We excluded all essays shorter than 50 characters, most of which were mentions to attachments (e.g., “See attached”). The critical role of extracurricular commitments (i.e., structured pursuits outside of the classroom) in the expression and development of personal qualities in youth has been documented in the literature on positive youth development (*45*, *46*).

#### Standardized test scores

Over half (55%) of the holdout sample reported SAT scores, 14% reported ACT scores, 25% reported both, and 6% reported neither. Using published guidelines (*47*), we converted ACT scores to SAT scores. For students who reported both test scores, we selected the higher score, and for students who reported neither, data were considered missing.

#### Extracurricular activities

Applicants listed up to seven extracurricular activities and for each, indicated the years they had participated. For each applicant, we computed the total number of extracurricular activities, mean years per activity, and the proportion of activities that were sports.

#### Demographics

We obtained the following demographic information from the Common Application: race/ethnicity, parental education, gender, parents’ marital status, English language learner status, and type of high school (i.e., title 1 public school versus other kinds of schools).

#### College graduation

We obtained data from the 2015 National Student Clearinghouse (NSC) database (www.studentclearinghouse.org) to create a binary 6-year graduation measure (0 = did not earn a bachelor’s degree from a 4-year institution within 6 years of initial enrollment; 1 = earned a bachelor’s within 6 years). We obtained institutional rates of graduation within 6 years from the National Center for Educational Statistics. We control for any potential effects of baseline institutional effects on the odds of graduation in the table S53.

### Analytic strategy

To handle missing data, we used multiple imputation (*m* = 25), using the mice package in R (*48*). We used predictive mean matching for graduation rates and college admissions test scores. For school type, we used polytomous regression. In the holdout sample, 5.7, 12.2, and 7.1% of students were missing data on admissions test scores, 6-year institutional graduation rates, and high school title 1 status, respectively.

In binary logistic regression models, we standardized all continuous variables to facilitate interpretation of ORs. Factor variables were dummy coded and, along with binary variables, were not standardized, such that the effects shown indicate the expected change in the odds of each variable relative to the comparison group. When averaging correlations together, we transformed the correlation coefficients to *z* scores using Fisher’s transformation, averaged them, and transformed them back to correlation coefficients.

Following convention, we report *P* values for our analyses. It is important to note that *P* values do not directly indicate practical importance, especially in the context of large sample sizes. With larger samples, even small effects can yield statistically significant results, potentially misleading interpretations of the findings. Therefore, we emphasize the importance of focusing on effect sizes, which provide a more meaningful measure of the magnitude of associations or differences.

### RoBERTa fine-tuning procedure

RoBERTa (*26*) is an advanced language representation model considered a meaningful innovation that improves on prior algorithms in the field of natural language processing. It is a deep neural network that has been pretrained by having it predict masked words in extremely large volumes of generic text (i.e., books and English Wikipedia). The fine-tuning process consists of adjusting the parameters of the final layers to maximize predictive accuracy in particular tasks (e.g., text classification) and in a particular corpus of text (e.g., admissions essays).

We used a subset of essays that were not manually coded to do a round of pretraining to optimize the RoBERTa model to our admission essay corpus. To do this, we trained RoBERTa to predict a masked word given the surrounding words. This process resulted in a RoBERTa model optimized for the particular prompt the essays in our corpus were answering. See section S2 for technical details on the pretraining process.

To begin the fine-tuning procedure, the second and third authors read random batches of 50 applicant essays to identify salient personal qualities commonly identified by colleges as desirable and/or shown in prior research to be related to positive life outcomes. After reading and discussing nine batches of 450 essays each, they developed criteria for seven personal qualities: prosocial purpose, leadership, teamwork, learning, perseverance, intrinsic motivation, and goal pursuit.

Next, we trained five research assistants to apply these criteria until each coder achieved adequate inter-rater reliability with either the second or third author across all seven attributes (Krippendorff’s α > 0.80). Raters then coded all 3131 essays in the development sample. Most of the essays were coded by a single rater (*n* = 2925; 93% of the development sample). To assess inter-rater reliability, pairs of raters independently coded a subset of essays (*n* = 206; 7% of the development sample).

In addition, we recruited 36 admissions officers to provide expert ratings of personal qualities. We recruited them through Character Collaborative, a mailing list sent by National Association for College Admission Counseling (NACAC), and the College Guidance Network. admissions officers completed a short training, which consisted on reading definitions, examples, and rating an example essay, and then were able to rate as many essays as they desired. Each admissions officer rated an average of 86 essays. Each essay in the development sample was rated by two different admissions officers.

We used these manually annotated datasets to fine-tune two sets of separate RoBERTa models to estimate the probability of each personal quality: one set on the ratings by research assistants and one set on the ratings by admission officers. After fine-tuning these models, we evaluated the performance of the models and applied it to the holdout sample of 306,463 essays, yielding more than two million continuous codes.

### Ethics statement

This research was approved by the University of Pennsylvannia IRB.

## Supplementary Materials

### This PDF file includes:

Sections 1 to 10 Figs. S1 to S7 Tables S1 to S53 References
