# Supplementary material for: Using artificial intelligence to assess personal qualities in college admissions
Source: Sci Adv. 2023 Oct 12;9(41):eadg9405. doi: 10.1126/sciadv.adg9405 (PMC10569720; doi:10.1126/sciadv.adg9405)
Supplement: Supplementary file 1 — Sections 1 to 10 Figs. S1 to S7 Tables S1 to S53 References [file sciadv.adg9405_sm.pdf]

Science Advances
AAAS

Supplementary Materials for

Using artificial intelligence to assess personal qualities in college admissions

Benjamin Lira *et al.*

Corresponding author: Benjamin Lira, blira@upenn.edu

Sci. Adv. **9**, eadg9405 (2023)
DOI: 10.1126/sciadv.adg9405

This PDF file includes:

Sections 1 to 10
Figs. S1 to S7
Tables S1 to S53
References

Section 1. Data and Exclusions

The dataset for this study emerged from a collaboration with the Common Application (Common App, www.commonapp.org) and the National Student Clearinghouse (NSC, www.studentclearinghouse.org). To protect privacy, Common App contracted a third-party organization to collect, anonymize, and deliver the dataset to our team. For additional details, see Hutt, et al. (*47*).

Specifically, our sample was drawn from the population of 413,675 students who completed the Common App during the 2008-09 academic year for college admission during the 2009-10 academic year. From this population, we selected the 311,308 students who had not enrolled in a postsecondary institution prior to 2008. This ensured the accuracy of records reflecting time to degree attainment.

Development Sample

Originally, we identified a stratified sample of applications for manual coding. As reported previously (*47*), we defined sampling strata based on the number of extracurricular activities reported on the Common Application as well as membership in one of five multi-dimensional demographic groups identified using latent class analysis (LCA). Specifically, our LCA model classified students according to profiles across race/ethnicity, parental education, parents' marital status, English language learner status (ELL), attended a Title 1 high school, and high school race/ethnic composition. The LCA was performed in MPlus 7 on the subset of all 213,091 students attending public schools. We excluded private and homeschooled students from this analysis because their school-level demographic data were not available. After excluding missing data, invalid responses, and essays coded by one rater who ultimately failed to achieve agreement with other raters, the *Development Sample* consisted of 3,131 students.

Holdout Sample

Of the original 311,308 applications, we were then left with the remaining 307,308 applications, which were not manually coded. We excluded 54 cases for which the algorithm failed to generate computer likelihoods, suggesting data errors; 786 essays with fewer than 50 characters (most of which had no content, e.g., "see attachment"); 3 applications with invalid essays (i.e., essays written by different applicants that were accidentally concatenated together); and 2 applications for which we had no available demographic information. This left us with a final *Holdout Sample* of 306,463 applicants. See **Figure S1** for a graphical representation of the sample composition.

Figure S1. Samples and exclusions. After all exclusions the *Development Sample* comprised 3,131 students, and the *Holdout Sample* comprised 306, 463 students.

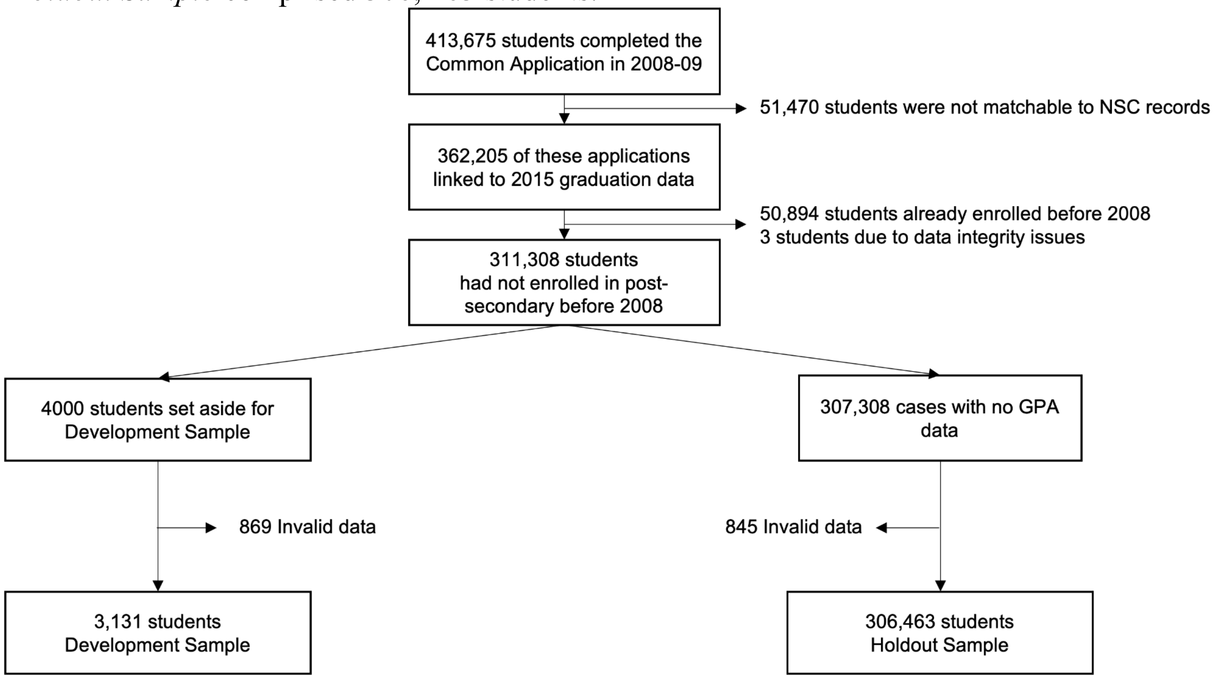

Section 2. RoBERTa Algorithm Fine-Tuning Procedure

We used the RoBERTa-base model, which we obtained from huggingface's "transformers" Python library. See this link to the model hosted on the huggingface website.

We began with pre-training, a procedure where the model is trained to identify words that have been removed from the text (i.e., masked language modeling). We used a single training epoch on unlabeled data to avoid overfitting.

We then finetuned the resulting model on our human-labelled dataset used 4 training epochs, with 32 examples used to predict on before updating the weights in each iteration (batch size = 32).

We used a 10-fold cross-validation procedure for training the model. Specifically, the *Development Sample* of 3,131 hand-coded essays was divided into 10 random subsets. We fine-tuned RoBERTa models on nine subsets and generated predictions on the held-out subset. We repeated this process until each subset was used for testing once. We then pooled the computer-generated likelihoods over the 10 iterations. All measures of model accuracy are based on out-of-sample predictions.

We used a binary classification framework. Specifically, we separately fine-tuned 10 models (one for each subset of cross-validation) for each of the seven personal qualities. Our final RoBERTa procedure entails applying these 70 RoBERTa models to each application essay and pooling predictions from each of the models to generate seven computer-generated likelihoods of personal qualities, which we used in subsequent analyses.

Section 3. Descriptive Statistics

Tables S1 through **S4** show descriptive statistics and correlations for the study variables in the *Development* and *Holdout Samples*, both for research assistants and admissions officers.

Table S1. Correlations and descriptive statistics in the *Development Sample – research assistants*

Variable	1	2	3	4	5	6	7	8	9	10	11	12
1. Prosocial purpose		.00	-.04*	-.08***	-.11***	-.04*	.05**	.00	.09***	.00	-.13***	.02
2. Leadership	-.02		.15***	-.03	-.01	-.07***	.04*	.06***	.11***	.11***	.05*	.07***
3. Teamwork	-.08***	.19***		.05**	.07***	-.02	.03	.05*	.06***	.03	.01	.02
4. Learning	-.11***	-.02	.06***		.08***	-.02	.01	.02	.07***	.02	.02	.05**
5. Perseverance	-.17***	.00	.05**	.13***		.04	.03	.07***	.06***	.05**	.02	.02
6. Intrinsic motivation	-.06**	-.11***	.00	-.03	.06**		.02	.02	-.02	.01	.04*	.01
7. Goal pursuit	.05**	.07***	.07***	-.05*	.05**	.04*		.04*	.08***	.06**	.02	.03
8. Standardized test scores	-.01	.08***	.05**	.02	.08***	.00	.03		.36***	.26***	-.11***	.30***
9. Number of activities	.09***	.13***	.11***	.06***	.07***	-.01	.08***	.36***		.40***	-.06**	.22***
10. Time per activity	-.01	.13***	.05**	.01	.06**	.05**	.07***	.26***	.40***		.28***	.17***
11. Proportion sports	-.12***	.03	.04*	.01	.05**	.08***	.03	-.11***	-.06**	.28***		.00
12. College graduation	.03	.07***	.05**	.06***	.03	.02	.03	.30***	.22***	.17***	.00	
M	0.36	0.19	0.26	0.45	0.19	0.45	0.32	1,693	3.46	2.11	0.23	0.66
SD	0.46	0.37	0.39	0.47	0.35	0.45	0.40	306	2.29	1.13		
N	3,120	3,124	3,103	3,126	3,125	3,116	3,124	2,834	3,131	3,131	3,131	3,131

Note. Correlations with computer-generated scores of personal qualities below the diagonal, correlations with human ratings of personal qualities above the diagonal.
*** $p < .001$. ** $p < .01$. * $p < .05$.

Table S2. Correlations and descriptive statistics in the *Development Sample – admissions officers*

Variable	1	2	3	4	5	6	7	8	9	10	11	12
1. Prosocial purpose		.12***	-.12***	-.21***	-.19***	-.06***	-.16***	.01	.12***	.01	-.13***	.05**
2. Leadership	.15***		.14***	-.14***	-.05**	-.16***	.01	.05**	.14***	.12***	.01	.06***
3. Teamwork	-.20***	.22***		-.04*	.11***	-.03†	.14***	.05**	.08***	.10***	.12***	.04*
4. Learning	-.32***	-.19***	-.08***		.03	-.03†	.09***	.07***	.03†	-.04*	-.12***	.03†
5. Perseverance	-.42***	-.11***	.16***	.14***		.03†	.24***	.05*	.03†	.08***	.10***	.04*
6. Intrinsic motivation	-.11***	-.30***	-.10***	.01	.12***		.07***	.09***	.06***	.06***	.03†	.07***
7. Goal pursuit	-.36***	-.04*	.16***	.22***	.45***	.07***		.04†	.05*	.09***	.07***	.03
8. Standardized test scores	-.01	.07***	.05**	.08***	.05**	.09***	.04†		.36***	.26***	-.11***	.30***
9. Number of activities	.13***	.18***	.10***	.06**	.03†	.04*	.07***	.36***		.40***	-.06**	.22***
10. Time per activity	.00	.13***	.13***	-.03†	.12***	.08***	.13***	.26***	.40***		.28***	.17***
11. Proportion sports	-.15***	.02	.19***	-.15***	.17***	.06**	.13***	-.11***	-.06**	.28***		.00
12. College graduation	.05*	.08***	.06**	.05**	.03†	.06***	.04*	.30***	.22***	.17***	.00	
M	0.30	0.25	0.22	0.46	0.24	0.42	0.25	1693	3.46	2.11	0.23	0.66
SD	0.37	0.33	0.28	0.33	0.25	0.28	0.24	306	2.29	1.13		
N	3,131	3,131	3,131	3,131	3,131	3,131	3,131	2,834	3,131	3,131	3,131	3,131

Note. Correlations with computer-generated scores of personal qualities below the diagonal, correlations with human ratings of personal qualities above the diagonal.
*** $p < .001$. ** $p < .01$. * $p < .05$.

Table S3. Correlations and descriptive statistics in the *Holdout Sample – research assistants*

Variable	1	2	3	4	5	6	7	8	9	10	11	12
1. Prosocial purpose												
2. Leadership	-.02***											
3. Teamwork	-.14***	.19***										
4. Learning	-.13***	-.03***	.06***									
5. Perseverance	-.18***	-.02***	.07***	.12***								
6. Intrinsic motivation	-.08***	-.12***	-.01***	-.06***	.04***							
7. Goal pursuit	.08***	.07***	-.01***	-.08***	.01***	.02***						
8. Standardized test scores	.00*	.07***	.06***	.00*	.07***	.02***	.05***					
9. Number of activities	.10***	.09***	.06***	.03***	.05***	.03***	.08***	.34***				
10. Time per activity	-.03***	.07***	.02***	-.01**	.02***	.06***	.00	.14***	.03***			
11. Proportion sports	-.10***	-.03***	.02***	.01***	.05***	.03***	-.03***	-.21***	-.27***	.09***		
12. College graduation	.04***	.05***	.04***	.02***	.02***	.02***	.02***	.22***	.18***	.09***	-.05***	
M	0.37	0.20	0.30	0.47	0.21	0.51	0.36	1,826	5.16	2.53	0.26	0.78
SD	0.46	0.37	0.39	0.46	0.35	0.44	0.40	267	1.98	0.76		

Note. *** $p < .001$. ** $p < .01$. * $p < .05$. $N = 306{,}463$ for all variables other than standardized test scores ($n = 289{,}140$)

Table S4. Correlations and descriptive statistics in the *Holdout Sample – admissions officers*

Variable	1	2	3	4	5	6	7	8	9	10	11	12
1. Prosocial purpose												
2. Leadership	.16***											
3. Teamwork	-.25***	.16***										
4. Learning	-.36***	-.22***	-.12***									
5. Perseverance	-.48***	-.15***	.19***	.12***								
6. Intrinsic motivation	-.14***	-.35***	-.12***	.03***	.14***							
7. Goal pursuit	-.42***	-.07***	.15***	.21***	.51***	.09***						
8. Standardized test scores	.00	.06***	.03***	.10***	.03***	.08***	.03***					
9. Number of activities	.13***	.12***	.02***	.06***	-.04***	.05***	.01***	.34***				
10. Time per activity	-.03***	.06***	.07***	-.04***	.07***	.08***	.05***	.14***	.03***			
11. Proportion sports	-.12***	-.04***	.14***	-.15***	.15***	-.01***	.08***	-.21***	-.27***	.09***		
12. College graduation	.04***	.06***	.04***	.01***	.01***	.02***	.02***	.22***	.18***	.09***	-.05***	
M	0.31	0.27	0.25	0.47	0.26	0.46	0.27	1,826	5.16	2.53	26.73%	77.83%
SD	0.37	0.33	0.28	0.31	0.24	0.26	0.23	267	1.98	0.76		

Note. *** $p < .001$. ** $p < .01$. * $p < .05$. $N = 306{,}463$ for all variables other than standardized test scores ($n = 289{,}140$)

Section 4. Relationship Between Personal Qualities and Demographics

As shown in **Table S5** and **S8,** demographic subgroup differences in the binary human ratings of personal qualities were small in magnitude (and in most cases not reliably different from zero) in the *Development Sample*, both for research assistants and admissions officers, respectively. As shown in **Table S6, S7, S9,** and **S10**, these differences were likewise small for the continuous computer-generated likelihoods of personal qualities in the *Development Sample* and *Holdout Sample*.

Table S5. Human ratings of personal qualities by demographic subgroup in the *Development Sample – research assistants*

Demographic variable	PP	LD	TW	LR	PS	IM	GP
Race/ethnicity							
White	−0.04	0.05	0.02	−0.02	0.06	0.07	0.05
Black	0.01	0.01	0.01	−0.02	−0.03	−0.05	0.03
Latino	0.05	0.00	−0.01	−0.03	0.00	−0.01	−0.01
Asian	0.03	−0.04	−0.02	0.06	−0.01	−0.04	−0.05
Other	0.01	0.01	−0.02	−0.02	−0.05	0.00	−0.02
Missing	−0.06	−0.05	0.01	0.03	0.00	0.02	0.00
Number of parents with college degrees							
None	0.03	0.02	−0.05	−0.05	−0.06	−0.08	−0.03
One	−0.02	−0.04	0.01	0.01	0.02	0.01	0.00
Two	−0.02	0.01	0.05	0.05	0.06	0.08	0.04
Female	0.12	0.03	−0.02	0.02	−0.01	0.02	−0.03
Married parents	−0.02	0.00	0.02	0.03	0.06	0.02	0.03
English language learner	0.04	−0.02	−0.03	0.02	0.00	−0.05	−0.04
Title 1 High School	0.00	0.02	0.00	−0.01	−0.03	−0.01	−0.01

Note. PP, Prosocial purpose; LD, Leadership; TW, Teamwork; LR, Learning; PS, Perseverance; IM, Intrinsic motivation; GP, Goal pursuit. Values are Matthew's correlation coefficients (phi)

Table S6. Computer-generated likelihoods of personal qualities by demographic subgroup in the *Development Sample – research assistants*

Demographic variable	PP	LD	TW	LR	PS	IM	GP
Race/ethnicity							
White	−0.12	0.13	0.13	−0.03	0.16	0.14	0.12
Black	0.02	−0.03	−0.06	−0.13	−0.15	−0.13	−0.04
Latino	0.11	0.00	0.00	−0.07	−0.02	−0.03	−0.01
Asian	0.14	−0.11	−0.09	0.20	−0.03	−0.12	−0.09
Other	0.05	0.06	−0.10	−0.08	−0.19	−0.04	−0.09
Missing	−0.18	−0.11	0.05	0.10	0.10	0.14	0.05
Number of parents with college degrees							
None	0.09	−0.02	−0.15	−0.13	−0.13	−0.19	−0.12
One	−0.08	−0.03	0.10	0.05	0.03	0.10	0.04
Two	−0.05	0.05	0.10	0.13	0.13	0.15	0.11
Female	0.28	0.06	−0.02	0.03	−0.06	0.07	−0.10
Married parents	0.00	0.03	0.04	0.03	0.07	0.06	0.10
English language learner	0.14	−0.10	−0.11	0.07	−0.09	−0.10	−0.07
Title 1 High School	0.03	0.04	0.00	−0.07	−0.04	−0.05	−0.02

Note. PP, Prosocial purpose; LD, Leadership; TW, Teamwork; LR, Learning; PS, Perseverance; IM, Intrinsic motivation; GP, Goal pursuit. Values are Cohen's *d*s

Table S7. Computer-generated likelihoods of personal qualities by demographic subgroup in the *Holdout Sample – research assistants*

Demographic variable	PP	LD	TW	LR	PS	IM	GP
Race/ethnicity							
White	−0.05	0.05	0.12	−0.08	0.08	0.04	0.05
Black	0.03	−0.02	−0.12	−0.12	−0.17	−0.18	−0.10
Latino	0.10	−0.03	−0.10	−0.04	−0.11	−0.08	−0.06
Asian	0.08	−0.03	−0.12	0.22	−0.02	−0.07	−0.03
Other	0.01	−0.01	−0.06	0.01	−0.03	0.04	0.00
Missing	−0.03	−0.03	−0.01	0.05	0.00	0.06	−0.01
Number of parents with college degrees							
None	0.00	−0.06	−0.09	−0.04	−0.10	−0.08	−0.08
One	−0.01	−0.01	−0.01	0.00	−0.03	0.00	0.00
Two	0.01	0.06	0.08	0.03	0.10	0.06	0.07
Female	0.22	0.06	0.01	0.03	0.02	0.14	0.03
Married parents	0.05	0.06	0.05	0.03	0.06	0.02	0.05
English language learner	0.06	−0.06	−0.05	0.05	0.06	−0.11	0.06
Title 1 high school	−0.02	0.02	−0.01	0.00	−0.03	−0.06	−0.02

Note. PP, Prosocial purpose; LD, Leadership; TW, Teamwork; LR, Learning; PS, Perseverance; IM, Intrinsic motivation; GP, Goal pursuit. Values are Cohen's *d*s

Table S8. Human ratings of personal qualities by demographic subgroup in the *Development Sample – admissions officers*

Demographic variable	PP	LD	TW	LR	PS	IM	GP
Race/ethnicity							
White	−0.04	0.05	0.02	−0.02	0.06	0.07	0.05
Black	0.01	0.01	0.01	−0.02	−0.03	−0.05	0.03
Latino	0.05	0.00	−0.01	−0.03	0.00	−0.01	−0.01
Asian	0.03	−0.04	−0.02	0.06	−0.01	−0.04	−0.05
Other	0.01	0.01	−0.02	−0.02	−0.05	0.00	−0.02
Missing	−0.06	−0.05	0.01	0.03	0.00	0.02	0.00
Number of parents with college degrees							
None	−0.04	0.05	0.02	−0.02	0.06	0.07	0.05
One	0.01	0.01	0.01	−0.02	−0.03	−0.05	0.03
Two	0.05	0.00	−0.01	−0.03	0.00	−0.01	−0.01
Female	0.03	−0.04	−0.02	0.06	−0.01	−0.04	−0.05
Married parents	0.01	0.01	−0.02	−0.02	−0.05	0.00	−0.02
English language learner	−0.06	−0.05	0.01	0.03	0.00	0.02	0.00
Title 1 High School	−0.04	0.05	0.02	−0.02	0.06	0.07	0.05

Note. PP, Prosocial purpose; LD, Leadership; TW, Teamwork; LR, Learning; PS, Perseverance; IM, Intrinsic motivation; GP, Goal pursuit. Values are Matthew's correlation coefficients (phi)

Table S9. Computer-generated likelihoods of personal qualities by demographic subgroup in the *Development Sample* – *admissions officers*

Demographic variable	PP	LD	TW	LR	PS	IM	GP
Race/ethnicity							
White	−0.12	0.13	0.13	−0.03	0.16	0.14	0.12
Black	0.02	−0.03	−0.06	−0.13	−0.15	−0.13	−0.04
Latino	0.11	0.00	0.00	−0.07	−0.02	−0.03	−0.01
Asian	0.14	−0.11	−0.09	0.20	−0.03	−0.12	−0.09
Other	0.05	0.06	−0.10	−0.08	−0.19	−0.04	−0.09
Missing	−0.18	−0.11	0.05	0.10	0.10	0.14	0.05
Number of parents with college degrees							
None	0.09	−0.02	−0.15	−0.13	−0.13	−0.19	−0.12
One	−0.08	−0.03	0.10	0.05	0.03	0.10	0.04
Two	−0.05	0.05	0.10	0.13	0.13	0.15	0.11
Female	0.28	0.06	−0.02	0.03	−0.06	0.07	−0.10
Married parents	0.00	0.03	0.04	0.03	0.07	0.06	0.10
English language learner	0.14	−0.10	−0.11	0.07	−0.09	−0.10	−0.07
Title 1 High School	0.03	0.04	0.00	−0.07	−0.04	−0.05	−0.02

Note. PP, Prosocial purpose; LD, Leadership; TW, Teamwork; LR, Learning; PS, Perseverance; IM, Intrinsic motivation; GP, Goal pursuit. Values are Cohen's *d*s

Table S10. Computer-generated likelihoods of personal qualities by demographic subgroup in the *Holdout Sample* – *admissions officers*

Demographic variable	PP	LD	TW	LR	PS	IM	GP
Race/ethnicity							
White	−0.05	0.05	0.12	−0.08	0.08	0.04	0.05
Black	0.03	−0.02	−0.12	−0.12	−0.17	−0.18	−0.10
Latino	0.10	−0.03	−0.10	−0.04	−0.11	−0.08	−0.06
Asian	0.08	−0.03	−0.12	0.22	−0.02	−0.07	−0.03
Other	0.01	−0.01	−0.06	0.01	−0.03	0.04	0.00
Missing	−0.03	−0.03	−0.01	0.05	0.00	0.06	−0.01
Number of parents with college degrees							
None	0.01	−0.08	−0.08	−0.10	−0.07	−0.15	−0.07
One	−0.01	−0.01	0.01	−0.04	0.00	−0.01	0.01
Two	0.00	0.07	0.06	0.10	0.05	0.12	0.05
Female	0.23	0.08	−0.05	0.01	−0.07	0.10	−0.07
Married parents	0.04	0.07	0.06	0.02	0.05	0.02	0.04
English language learner	0.07	−0.08	−0.17	0.17	−0.08	−0.08	−0.05
Title 1 high school	−0.02	0.01	0.00	−0.01	−0.02	−0.08	0.02

Note. PP, Prosocial purpose; LD, Leadership; TW, Teamwork; LR, Learning; PS, Perseverance; IM, Intrinsic motivation; GP, Goal pursuit. Values are Cohen's *d*s

Section 5. Human-Computer Correlations Across Demographic Subgroups

As shown in **Tables S11** and **S12,** the convergent validity for each group was, for the most part, not significantly different from the convergent validity of the most populated subgroup.

Table S11. Correlations between human ratings and computer-generated likelihoods of personal qualities by demographic subgroup in the Development Sample – research assistants

Demographic category	*n*	PP	LD	TW	LR	PS	IM	GP	ACV	ADV	Range of DV	
Race/ethnicity												
White	871	0.87	0.79	0.59	0.80	0.68	0.73	0.58	0.74	-0.01	-0.15	0.14
Black	487	0.84	0.78	0.64	0.76	0.72	0.78	0.60	0.74	0.00	-0.27	0.22
Latino	501	0.86	0.85	0.60	0.78	0.63	0.71	0.67	0.74	0.02	-0.14	0.21
Asian	590	0.84	0.80	0.62	0.72	0.67	0.70	0.54	0.71	0.01	-0.15	0.20
Other	290	0.84	0.83	0.61	0.72	0.56	0.74	0.54	0.71	0.01	-0.21	0.23
No race reported	369	0.90	0.85	0.66	0.77	0.70	0.75	0.65	0.77	0.01	-0.14	0.20
Parents with college degrees												
None	1,608	0.85	0.81	0.60	0.78	0.66	0.75	0.61	0.74	0.01	-0.19	0.20
One	653	0.86	0.81	0.62	0.79	0.65	0.71	0.57	0.73	0.01	-0.12	0.17
Two	853	0.88	0.80	0.63	0.74	0.69	0.71	0.58	0.73	0.00	-0.14	0.19
Gender												
Female	1,702	0.86	0.81	0.62	0.77	0.68	0.73	0.60	0.74	0.00	-0.18	0.18
Male	1,413	0.85	0.81	0.61	0.77	0.66	0.74	0.58	0.73	0.00	-0.15	0.17
Married parents												
Parents married	2,055	0.85	0.81	0.61	0.77	0.68	0.74	0.58	0.73	0.00	-0.15	0.17
Parents not married	1,061	0.88	0.80	0.63	0.77	0.66	0.71	0.62	0.74	0.01	-0.18	0.19
English language learner status												
English language learner	808	0.87	0.77	0.59	0.74	0.69	0.72	0.61	0.73	0.01	-0.15	0.17
Native speaker	2,308	0.86	0.82	0.62	0.78	0.66	0.73	0.59	0.74	0.00	-0.17	0.18
Title 1 status of high school												
Title 1 public school	1,127	0.83	0.84	0.61	0.77	0.67	0.74	0.59	0.74	0.01	-0.21	0.21
Non-Title 1 school	1,552	0.88	0.80	0.63	0.78	0.66	0.72	0.60	0.74	0.00	-0.12	0.16

Note. All correlations are point-biserial correlation coefficients between binary human ratings and continuous computer-generated likelihoods. All correlations were significantly different from zero ($p < .001$). ACV (average convergent validities) are the average correlations between human ratings and computer-generated likelihoods for the same personal qualities. ADV (average discriminant validities) are the average correlations between human ratings and computer-generated likelihoods for differing personal qualities. n = 3,131.

Table S12, Correlations between human ratings and computer-generated likelihoods of personal qualities by demographic subgroup in the Development Sample – admissions officers

	n	PP	LD	TW	LR	PS	IM	GP	CV	ADV	Range of DV	
**Race/ethnicity**												
White	875	0.80	0.73	0.60	0.61	0.51	0.42	0.47	0.61	−0.02	−0.24	0.10
Black	489	0.80	0.71	0.64	0.68	0.46	0.48	0.43	0.62	−0.03	−0.27	0.14
Latino	503	0.82	0.77	0.67	0.68	0.49	0.43	0.41	0.63	−0.01	−0.25	0.15
Asian	591	0.80	0.72	0.63	0.66	0.52	0.44	0.43	0.62	−0.01	−0.24	0.13
Other	291	0.73	0.74	0.54	0.64	0.31	0.46	0.47	0.57	−0.02	−0.22	0.18
No race reported	370	0.82	0.72	0.63	0.65	0.44	0.49	0.46	0.62	0.00	−0.21	0.13
**Number of parents with college degrees**												
None	1,613	0.80	0.75	0.63	0.68	0.43	0.45	0.41	0.61	−0.01	−0.21	0.12
One	655	0.77	0.70	0.63	0.63	0.51	0.46	0.51	0.61	−0.03	−0.26	0.15
Two	857	0.84	0.73	0.60	0.60	0.55	0.42	0.46	0.62	−0.02	−0.27	0.11
**Female**												
Female	1,707	0.81	0.73	0.62	0.65	0.49	0.44	0.44	0.62	−0.01	−0.25	0.12
Male	1,420	0.78	0.74	0.62	0.65	0.48	0.46	0.45	0.61	−0.02	−0.23	0.09
**Married parents**												
Parents married	2,063	0.79	0.74	0.60	0.65	0.50	0.44	0.46	0.61	−0.02	−0.25	0.11
Parents not married	1,064	0.82	0.73	0.67	0.65	0.45	0.46	0.40	0.62	−0.01	−0.22	0.14
**English language learner**												
Learner	811	0.80	0.73	0.64	0.67	0.48	0.43	0.38	0.61	−0.03	−0.19	0.11
Native Speaker	2,316	0.80	0.73	0.61	0.64	0.48	0.45	0.46	0.62	−0.01	−0.26	0.11
**Title-I high school**												
Title-I Public School	1,131	0.79	0.79	0.63	0.67	0.45	0.47	0.44	0.63	−0.02	−0.22	0.12
Non-Title-I School	1,558	0.81	0.70	0.61	0.63	0.50	0.44	0.47	0.61	−0.02	−0.26	0.14

Note. All correlations are point-biserial correlation coefficients between binary human ratings and continuous computer-generated likelihoods. All correlations were significantly different from zero ($p < .001$). ACV (average convergent validities) are the average correlations between human ratings and computer-generated likelihoods for the same personal qualities. ADV (average discriminant validities) are the average correlations between human ratings and computer-generated likelihoods for differing personal qualities. *n* = 3,131.

Table S13 and S14 shows the correlation between human ratings and computer-generated likelihoods of personal qualities for each subgroup compared to the reference group. In most cases the difference between these correlations were not significant both for research assistants and admissions officers.

Table S13. Difference between human-computer correlations for each subgroup compared to the reference group – research assistants

	PP			LD			TW			LR			PS			IM			GP		
	C	R	D	C	R	D	C	R	D	C	R	D	C	R	D	C	R	D	C	R	D
Race/ethnicity (vs. White)																					
Black	.84	.87	-.04	.78	.79	-.01	.64	.59	.05	.76	.80	-.04	.72	.68	.04	.78	.73	.05	.60	.58	.01
Latino	.86	.87	-.02	.85	.79	.06*	.60	.59	.02	.78	.80	-.03	.63	.68	-.05	.71	.73	-.02	.67	.58	.08
Asian	.84	.87	-.03	.80	.79	.01	.62	.59	.03	.72	.80	-.08**	.67	.68	-.01	.70	.73	-.02	.54	.58	-.04
Other	.84	.87	-.03	.83	.79	.04	.61	.59	.02	.72	.80	-.08*	.56	.68	-.12*	.74	.73	.01	.54	.58	-.05
No race reported	.90	.87	.02	.85	.79	.06*	.66	.59	.07	.77	.80	-.04	.70	.68	.02	.75	.73	.02	.65	.58	.06
Number of parents with college degrees (vs. None)																					
One	.86	.85	.01	.81	.81	-.00	.62	.60	.02	.79	.78	.01	.65	.66	-.01	.71	.75	-.04	.57	.61	-.04
Two	.88	.85	.03*	.80	.81	-.01	.63	.60	.02	.74	.78	-.04	.69	.66	.03	.71	.75	-.04	.58	.61	-.04
Other demographics																					
Male	.85	.86	-.01	.81	.81	.00	.61	.62	-.01	.77	.77	-.00	.66	.68	-.02	.74	.73	.01	.58	.60	-.02
Parents not married	.88	.85	.03*	.80	.81	-.02	.63	.61	.02	.77	.77	.01	.66	.68	-.02	.71	.74	-.02	.62	.58	.04
English language learner	.87	.86	.02	.77	.82	-.05*	.59	.62	-.03	.74	.78	-.03	.69	.66	.02	.72	.73	-.02	.61	.59	.02
Title 1 public school	.88	.83	.04**	.80	.84	-.04*	.63	.61	.02	.78	.77	.01	.66	.67	-.01	.72	.74	-.02	.60	.59	.01

Note. PP, Prosocial purpose; LD, Leadership; TW, Teamwork; LR, Learning; PS, Perseverance; IM, Intrinsic motivation; GP, Goal pursuit. C, Human-computer correlation for the comparison group; R, Human-computer correlation for the reference group; D, Differences between point-biserial correlations. *p*-values are adjusted for multiple comparisons using the Benjamini Hochberg False Discovery Rate correction (27).

Table S14. Difference between human-computer correlations for each subgroup compared to the reference group – admissions officers

	PP			LD			TW			LR			PS			IM			GP		
	C	R	D	C	R	D	C	R	D	C	R	D	C	R	D	C	R	D	C	R	D
Race/ethnicity (vs. White)																					
Black	.80	.80	-.00	.71	.73	-.01	.64	.60	.04	.68	.61	.07	.46	.51	-.05	.48	.42	.06	.43	.47	-.04
Latino	.82	.80	.01	.77	.73	.04	.67	.60	.07	.68	.61	.07	.49	.51	-.03	.43	.42	.01	.41	.47	-.06
Asian	.80	.80	-.00	.72	.73	-.01	.63	.60	.03	.66	.61	.04	.52	.51	.01	.44	.42	.02	.43	.47	-.04
Other	.73	.80	-.07	.74	.73	.02	.54	.60	-.06	.64	.61	.03	.31	.51	-<br>.21**	.46	.42	.04	.47	.47	-.00
No race reported	.82	.80	.02	.72	.73	-.01	.63	.60	.03	.65	.61	.04	.44	.51	-.07	.49	.42	.07	.46	.47	-.01
Number of parents with college degrees (vs. None)																					
One	.77	.80	-.02	.70	.75	-.04	.63	.63	.00	.63	.68	-.05	.51	.43	.08	.46	.45	.01	.51	.41	.11*
Two	.84	.80	.04*	.73	.75	-.02	.60	.63	-.03	.60	.68	-.08*	.55	.43	.12**	.42	.45	-.04	.46	.41	.06
Other demographics																					
Male	.78	.81	-.03	.74	.73	.01	.62	.62	.01	.65	.65	-.01	.48	.49	-.01	.46	.44	.01	.45	.44	.01
Parents not married	.82	.79	.02	.73	.74	-.01	.67	.60	.07*	.65	.65	.00	.45	.50	-.05	.46	.44	.02	.40	.46	-.06
English language learner	.80	.80	.00	.73	.73	.00	.64	.61	.03	.67	.64	.02	.48	.48	-.00	.43	.45	-.02	.38	.46	-.08
Title 1 public school	.79	.81	-.02	.79	.70	.09***	.63	.61	.02	.67	.63	.04	.45	.50	-.05	.47	.44	.03	.44	.47	.04

Note. PP, Prosocial purpose; LD, Leadership; TW, Teamwork; LR, Learning; PS, Perseverance; IM, Intrinsic motivation; GP, Goal pursuit. C, Human-computer correlation for the comparison group; R, Human-computer correlation for the reference group; D, Differences between point-biserial correlations. *p*-values are adjusted for multiple comparisons using the Benjamini Hochberg False Discovery Rate correction (*27*).

Tables S15 to **S31** show descriptive statistics and point biserial correlations between computer-generated likelihoods (rows) and human ratings (columns) of personal qualities for each of 17 subgroups defined by personal characteristics (i.e., gender, parental education, parental marital status, English language learner status, race/ethnicity, and type of high school), for research assistants. **Tables S32** to **S48** show the equivalent information for admission officer ratings and computer-generated likelihoods.

Table S15. Descriptive statistics and point biserial correlations between computer-generated likelihoods and human ratings of personal qualities in the *Development Sample* for White applicants – research assistants

Personal quality	1	2	3	4	5	6	7
Computer-generated likelihoods							
1. Prosocial purpose	.87***	-.03	-.07*	-.08*	-.11***	-.04	.07*
2. Leadership	-.04	.79***	.08*	-.04	-.03	-.13***	.01
3. Teamwork	-.10**	.11***	.59***	.07*	.04	-.01	.03
4. Learning	-.15***	-.04	.04	.80***	.14***	-.04	-.07*
5. Perseverance	-.15***	-.07*	.05	.09**	.68***	.04	.06
6. Intrinsic motivation	-.04	-.13***	.04	-.05	.04	.73***	.02
7. Goal pursuit	.10**	-.01	.02	.01	.00	.04	.58***
Frequency of human rating	0.31	0.20	0.26	0.42	0.19	0.44	0.34
Mean of computer-generated likelihood	0.33	0.22	0.27	0.44	0.19	0.50	0.34

Note. *** $p < .001$. ** $p < .01$. * $p < .05$.

Table S16. Descriptive statistics and point biserial correlations between computer-generated likelihoods and human ratings of personal qualities in the *Development Sample* for Black applicants – research assistants

Personal quality	1	2	3	4	5	6	7
Computer-generated likelihoods							
1. Prosocial purpose	.84***	-.04	.03	-.06	-.22***	-.04	-.03
2. Leadership	.04	.78***	.18***	-.07	-.08	-.06	.06
3. Teamwork	.05	.22***	.64***	.02	.02	-.02	.07
4. Learning	-.06	-.04	.03	.76***	.04	-.02	-.03
5. Perseverance	-.27***	-.09*	.06	.09	.72***	.01	-.02
6. Intrinsic motivation	-.09*	-.02	.01	-.07	.05	.78***	.10*
7. Goal pursuit	.00	.03	.15**	-.03	-.02	.02	.60***
Frequency of human rating	0.34	0.16	0.22	0.38	0.14	0.40	0.32
Mean of computer-generated likelihood	0.36	0.18	0.23	0.42	0.15	0.44	0.33

Note. *** $p < .001$. ** $p < .01$. * $p < .05$.

Table S17. Descriptive statistics and point biserial correlations between computer-generated likelihoods and human ratings of personal qualities in the *Development Sample* for Latino applicants – research assistants

Personal quality	1	2	3	4	5	6	7
Computer-generated likelihoods							
1. Prosocial purpose	.86***	.03	-.02	-.14**	-.13**	-.04	.06
2. Leadership	.00	.85***	.19***	-.02	.10*	.01	.10*
3. Teamwork	-.09*	.21***	.60***	.08	.17***	.00	.10*
4. Learning	-.12**	-.07	.09*	.78***	.10*	.00	-.01
5. Perseverance	-.12**	.10*	.08	.10*	.63***	.05	.08
6. Intrinsic motivation	-.02	-.05	-.06	.01	.02	.71***	-.01
7. Goal pursuit	.06	.10*	.08	-.08	.09*	.01	.67***
Frequency of human rating	0.37	0.18	0.27	0.41	0.22	0.41	0.28
Mean of computer-generated likelihood	0.39	0.18	0.27	0.44	0.20	0.44	0.32

Note. *** $p < .001$. ** $p < .01$. * $p < .05$.

Table S18. Descriptive statistics and point biserial correlations between computer-generated likelihoods and human ratings of personal qualities in the *Development Sample* for Asian applicants – research assistants

Personal quality	1	2	3	4	5	6	7
Computer-generated likelihoods							
1. Prosocial purpose	.84***	.03	-.11**	-.08*	-.07	-.04	.04
2. Leadership	-.02	.80***	.20***	.01	.02	-.15***	.05
3. Teamwork	-.15***	.18***	.62***	.06	.06	-.01	.07
4. Learning	-.08*	-.12**	.00	.72***	.08*	-.02	.00
5. Perseverance	-.14***	.04	.06	.09*	.67***	.11**	.11**
6. Intrinsic motivation	-.05	-.10*	-.01	-.05	.14***	.70***	-.02
7. Goal pursuit	.04	.16***	.10*	-.01	.05	.01	.54***
Frequency of human rating	0.40	0.16	0.30	0.47	0.22	0.38	0.32
Mean of computer-generated likelihood	0.41	0.18	0.28	0.50	0.21	0.39	0.32

Note. *** $p < .001$. ** $p < .01$. * $p < .05$.

Table S19. Descriptive statistics and point biserial correlations between computer-generated likelihoods and human ratings of personal qualities in the *Development Sample* for applicants reporting other races/ethnicities – research assistants

Personal quality	1	2	3	4	5	6	7
Computer-generated likelihoods							
1. Prosocial purpose	.84***	.03	-.04	-.16**	-.21***	-.05	-.01
2. Leadership	-.01	.83***	.14*	.05	.01	-.17**	.08
3. Teamwork	-.05	.23***	.61***	.01	.07	-.08	.08
4. Learning	-.07	-.03	.05	.72***	.08	.08	.07
5. Perseverance	-.15**	-.07	.06	.14*	.56***	-.08	.06
6. Intrinsic motivation	.06	-.15*	-.07	.09	-.10	.74***	.10
7. Goal pursuit	.06	.08	.04	.09	-.06	.03	.54***
Frequency of human rating	0.36	0.20	0.20	0.35	0.14	0.43	0.34
Mean of computer-generated likelihood	0.37	0.20	0.22	0.38	0.14	0.44	0.34

Note. *** $p < .001$. ** $p < .01$. * $p < .05$.

Table S20. Descriptive statistics and point biserial correlations between computer-generated likelihoods and human ratings of personal qualities in the *Development Sample* for applicants who did not report their race/ethnicity – research assistants

Personal quality	1	2	3	4	5	6	7
Computer-generated likelihoods							
1. Prosocial purpose	.90***	-.02	.02	-.07	-.09	-.10*	.11*
2. Leadership	.01	.85***	.20***	.06	.01	-.04	.02
3. Teamwork	.00	.20***	.66***	.05	.01	.00	.01
4. Learning	-.08	.02	-.02	.77***	.15**	.00	-.05
5. Perseverance	-.14**	.03	.00	.12*	.70***	-.02	.01
6. Intrinsic motivation	-.09	-.05	.02	-.07	-.02	.75***	.04
7. Goal pursuit	.11*	.04	-.02	-.03	.07	.01	.65***
Frequency of human rating	0.30	0.15	0.26	0.43	0.20	0.43	0.26
Mean of computer-generated likelihood	0.30	0.17	0.27	0.49	0.21	0.47	0.28

Note. *** $p < .001$. ** $p < .01$,.* $p < .05$.

Table S21. Descriptive statistics and point biserial correlations between computer-generated likelihoods and human ratings of personal qualities in the *Development Sample* for applicants with no parents with college degrees – research assistants

Personal quality	1	2	3	4	5	6	7
Computer-generated likelihoods							
1. Prosocial purpose	.85***	-.03	-.03	-.07**	-.13***	-.04	.05*
2. Leadership	-.02	.81***	.17***	.02	.02	-.07**	.06*
3. Teamwork	-.09***	.20***	.60***	.07**	.05	-.02	.03
4. Learning	-.09***	-.03	.07**	.78***	.08**	-.02	-.02
5. Perseverance	-.19***	.02	.05	.07**	.66***	.02	.03
6. Intrinsic motivation	-.03	-.07**	-.04	-.04	.02	.75***	.04
7. Goal pursuit	.05*	.07**	.06*	-.03	.03	.04	.61***
Frequency of human rating	0.36	0.17	0.24	0.40	0.17	0.42	0.30
Mean of computer-generated likelihood	0.38	0.19	0.23	0.44	0.18	0.45	0.32

Note. *** $p < .001$. ** $p < .01$. * $p < .05$.

Table S22. Descriptive statistics and point biserial correlations between computer-generated likelihoods and human ratings of personal qualities in the *Development Sample* for applicants with one parent with a college degree – research assistants

Personal quality	1	2	3	4	5	6	7
Computer-generated likelihoods							
1. Prosocial purpose	.86***	.05	-.07	-.09*	-.09*	-.03	.03
2. Leadership	.02	.81***	.07	-.05	-.04	-.11**	.05
3. Teamwork	-.05	.08*	.62***	.06	.09*	.04	.10*
4. Learning	-.06	-.07	.04	.79***	.13***	.01	-.05
5. Perseverance	-.12**	-.08*	.07	.17***	.65***	.01	.03
6. Intrinsic motivation	-.04	-.11**	.11**	-.04	.08*	.71***	.04
7. Goal pursuit	.07	.02	.03	.00	-.04	.00	.57***
Frequency of human rating	0.32	0.17	0.26	0.41	0.18	0.41	0.32
Mean of computer-generated likelihood	0.34	0.19	0.28	0.45	0.18	0.45	0.31

Note. *** $p < .001$. ** $p < .01$. * $p < .05$.

Table S23. Descriptive statistics and point biserial correlations between computer-generated likelihoods and human ratings of personal qualities in the *Development Sample* for applicants with two parents with college degrees – research assistants

Personal quality	1	2	3	4	5	6	7
Computer-generated likelihoods							
1. Prosocial purpose	.88***	-.01	-.04	-.12***	-.13***	-.09**	.04
2. Leadership	-.02	.80***	.18***	-.03	.00	-.11**	.02
3. Teamwork	-.03	.19***	.63***	.02	.06	-.06	.06
4. Learning	-.14***	-.07*	-.03	.74***	.15***	-.03	-.04
5. Perseverance	-.13***	-.02	.07	.11**	.69***	.07*	.10**
6. Intrinsic motivation	-.09**	-.10**	-.03	-.02	.05	.71***	.00
7. Goal pursuit	.08*	.07*	.08*	.01	.05	.01	.58***
Frequency of human rating	0.33	0.19	0.28	0.45	0.22	0.43	0.34
Mean of computer-generated likelihood	0.34	0.20	0.30	0.48	0.21	0.46	0.35

Note. *** $p < .001$. ** $p < .01$. * $p < .05$.

Table S24. Descriptive statistics and point biserial correlations between computer-generated likelihoods and human ratings of personal qualities in the *Development Sample* for female applicants – research assistants

Personal quality	1	2	3	4	5	6	7
Computer-generated likelihoods							
1. Prosocial purpose	.86***	.00	-.05*	-.10***	-.16***	-.02	.04
2. Leadership	.00	.81***	.16***	.00	.02	-.09***	.03
3. Teamwork	-.10***	.18***	.62***	.05*	.07**	-.03	.04
4. Learning	-.10***	-.04	.06*	.77***	.11***	-.02	-.02
5. Perseverance	-.18***	.00	.07**	.09***	.68***	.03	.05*
6. Intrinsic motivation	-.03	-.11***	-.01	-.05	.02	.73***	.02
7. Goal pursuit	.08**	.06*	.08***	-.01	.02	.03	.60***
Frequency of human rating	0.40	0.19	0.27	0.44	0.20	0.44	0.31
Mean of computer-generated likelihood	0.41	0.20	0.27	0.47	0.20	0.48	0.33

Note. *** $p < .001$. ** $p < .01$. * $p < .05$.

Table S25. Descriptive statistics and point biserial correlations between computer-generated likelihoods and human ratings of personal qualities in the *Development Sample* for male applicants – research assistants

Personal quality	1	2	3	4	5	6	7
Computer-generated likelihoods							
1. Prosocial purpose	.85***	-.02	-.04	-.10***	-.09***	-.11***	.04
2. Leadership	-.03	.81***	.14***	-.03	-.02	-.09***	.07**
3. Teamwork	-.05	.17***	.61***	.07*	.06*	-.01	.08**
4. Learning	-.12***	-.07*	.00	.77***	.11***	-.01	-.04
5. Perseverance	-.15***	-.04	.04	.12***	.66***	.03	.05
6. Intrinsic motivation	-.10***	-.06*	.00	-.03	.06*	.74***	.05
7. Goal pursuit	.03	.05	.03	-.01	.02	.01	.58***
Frequency of human rating	0.27	0.17	0.24	0.39	0.17	0.39	0.32
Mean of computer-generated likelihood	0.29	0.18	0.25	0.43	0.17	0.42	0.31

Note. *** $p < .001$. ** $p < .01$. * $p < .05$.

Table S26. Descriptive statistics and point biserial correlations between computer-generated likelihoods and human ratings of personal qualities in the *Development Sample* for applicants with married parents – research assistants

Personal quality	1	2	3	4	5	6	7
Computer-generated likelihoods							
1. Prosocial purpose	.85***	-.01	-.04*	-.12***	-.12***	-.04*	.04
2. Leadership	-.01	.81***	.15***	-.01	.02	-.09***	.05*
3. Teamwork	-.07**	.17***	.61***	.06**	.08***	.00	.05*
4. Learning	-.12***	-.06**	.02	.77***	.12***	-.03	-.03
5. Perseverance	-.15***	-.01	.05*	.10***	.68***	.03	.06**
6. Intrinsic motivation	-.06**	-.09***	.02	-.05*	.03	.74***	.02
7. Goal pursuit	.05*	.07**	.05*	-.02	.03	.01	.58***
Frequency of human rating	0.34	0.18	0.26	0.42	0.19	0.42	0.32
Mean of computer-generated likelihood	0.35	0.20	0.27	0.45	0.20	0.46	0.33

Note. *** $p < .001$. ** $p < .01$. * $p < .05$.

Table S27. Descriptive statistics and point biserial correlations between computer-generated likelihoods and human ratings of personal qualities in the *Development Sample* for applicants with parents who are not married – research assistants

Personal quality	1	2	3	4	5	6	7
Computer-generated likelihoods							
1. Prosocial purpose	.88***	.00	-.04	-.04	-.13***	-.06*	.05
2. Leadership	-.01	.80***	.15***	-.02	-.02	-.08**	.05
3. Teamwork	-.07*	.19***	.63***	.05	.04	-.04	.06*
4. Learning	-.07*	-.03	.07*	.77***	.08**	.01	-.02
5. Perseverance	-.18***	-.01	.07*	.11***	.66***	.05	.04
6. Intrinsic motivation	-.03	-.07*	-.04	-.01	.06	.71***	.06
7. Goal pursuit	.08**	.04	.08**	.01	.02	.04	.62***
Frequency of human rating	0.35	0.17	0.25	0.42	0.18	0.41	0.30
Mean of computer-generated likelihood	0.37	0.18	0.25	0.45	0.17	0.45	0.31

Note. *** $p < .001$. ** $p < .01$. * $p < .05$.

Table S28. Descriptive statistics and point biserial correlations between computer-generated likelihoods and human ratings of personal qualities in the *Development Sample* for English language learner applicants – research assistants

Personal quality	1	2	3	4	5	6	7
Computer-generated likelihoods							
1. Prosocial purpose	.87***	.05	-.05	-.14***	-.09**	-.03	.02
2. Leadership	.03	.77***	.13***	.00	.00	-.08*	.06
3. Teamwork	-.08*	.17***	.59***	.06	.10**	-.01	.07*
4. Learning	-.15***	-.09**	.06	.74***	.08*	.01	-.02
5. Perseverance	-.15***	.02	.05	.10**	.69***	.03	.09*
6. Intrinsic motivation	-.03	-.09*	-.01	-.01	.03	.72***	-.01
7. Goal pursuit	.02	.11**	.08*	-.02	.04	.01	.61***
Frequency of human rating	0.38	0.16	0.28	0.43	0.21	0.39	0.32
Mean of computer-generated likelihood	0.40	0.17	0.27	0.47	0.21	0.42	0.34

Note. *** $p < .001$. ** $p < .01$. * $p < .05$.

Table S29. Descriptive statistics and point biserial correlations between computer-generated likelihoods and human ratings of personal qualities in the *Development Sample* for native English-speaking applicants – research assistants

Personal quality	1	2	3	4	5	6	7
Computer-generated likelihoods							
1. Prosocial purpose	.86***	-.02	-.04	-.08***	-.14***	-.06**	.05*
2. Leadership	-.03	.82***	.16***	-.02	.00	-.10***	.05*
3. Teamwork	-.07**	.18***	.62***	.06**	.05*	-.02	.05*
4. Learning	-.08***	-.04	.03	.78***	.12***	-.02	-.03
5. Perseverance	-.17***	-.02	.06**	.10***	.66***	.04	.04
6. Intrinsic motivation	-.05**	-.09***	.00	-.04*	.04*	.73***	.05*
7. Goal pursuit	.07***	.04*	.05*	-.01	.02	.03	.59***
Frequency of human rating	0.33	0.18	0.25	0.41	0.18	0.43	0.31
Mean of computer-generated likelihood	0.34	0.20	0.26	0.45	0.18	0.46	0.32

Note. *** $p < .001$. ** $p < .01$. * $p < .05$.

Table S30. Descriptive statistics and point biserial correlations between computer-generated likelihoods and human ratings of personal qualities in the *Development Sample* for applicants who attended Title 1 public high schools – research assistants

Personal quality	1	2	3	4	5	6	7
Computer-generated likelihoods							
1. Prosocial purpose	.83***	.00	-.04	-.05	-.18***	-.05	.05
2. Leadership	.02	.84***	.19***	-.03	.05	-.07*	.06*
3. Teamwork	-.08*	.21***	.61***	.04	.12***	-.04	.07*
4. Learning	-.08**	-.06*	.03	.77***	.10***	-.01	-.06
5. Perseverance	-.21***	.02	.08**	.11***	.67***	.04	.06*
6. Intrinsic motivation	-.07*	-.11***	-.04	-.06	.03	.74***	.04
7. Goal pursuit	.05	.08**	.07*	-.04	.06*	.05	.59***
Frequency of human rating	0.35	0.20	0.25	0.41	0.18	0.43	0.30
Mean of computer-generated likelihood	0.36	0.21	0.27	0.44	0.19	0.47	0.31

Note. *** $p < .001$. ** $p < .01$. * $p < .05$.

Table S31. Descriptive statistics and point biserial correlations between computer-generated likelihoods and human ratings of personal qualities in the *Development Sample* for applicants who attended non-Title-1 high schools – research assistants

Personal quality	1	2	3	4	5	6	7
Computer-generated likelihoods							
1. Prosocial purpose	.88***	-.02	-.06*	-.12***	-.09***	-.05	.03
2. Leadership	-.03	.80***	.14***	.00	-.03	-.12***	.04
3. Teamwork	-.07**	.16***	.63***	.06*	.02	-.01	.06*
4. Learning	-.11***	-.03	.04	.78***	.13***	-.03	-.02
5. Perseverance	-.12***	-.02	.05*	.09***	.66***	.02	.04
6. Intrinsic motivation	-.03	-.08**	.02	-.02	.04	.72***	.03
7. Goal pursuit	.05	.05	.05*	.02	-.01	.01	.60***
Frequency of human rating	0.34	0.18	0.26	0.43	0.19	0.42	0.31
Mean of computer-generated likelihood	0.35	0.20	0.26	0.46	0.20	0.45	0.32

Note. *** $p < .001$. ** $p < .01$. * $p < .05$.

Table S32. Descriptive statistics and point biserial correlations between computer-generated likelihoods and human ratings of personal qualities in the *Development Sample* for White applicants – admissions officers

Personal quality	1	2	3	4	5	6	7
Computer-generated likelihoods							
1. Prosocial purpose	.82***	.16***	-.17***	-.24***	-.25***	-.06	-.15***
2. Leadership	.11*	.77***	.21***	-.22***	-.08	-.14**	.02
3. Teamwork	-.21***	.18***	.67***	-.13**	.09*	.02	.15***
4. Learning	-.27***	-.21***	-.11*	.68***	.07	.02	.12**
5. Perseverance	-.39***	-.06	.20***	.05	.49***	.09	.27***
6. Intrinsic motivation	-.06	-.20***	-.10*	.00	.03	.43***	.06
7. Goal pursuit	-.33***	-.02	.16***	.12**	.26***	.08	.41***
Frequency of human rating	0.65	0.49	0.44	0.81	0.43	0.80	0.48
Mean of computer-generated likelihood	0.33	0.25	0.22	0.44	0.24	0.41	0.25

Note. *** $p < .001$. ** $p < .01$. * $p < .05$.

Table S33. Descriptive statistics and point biserial correlations between computer-generated likelihoods and human ratings of personal qualities in the *Development Sample* for Black applicants – admissions officers

Personal quality	1	2	3	4	5	6	7
Computer-generated likelihoods							
1. Prosocial purpose	.80***	.19***	-.07	-.17***	-.27***	-.11*	-.20***
2. Leadership	.19***	.71***	.18***	-.14**	-.12*	-.09*	-.06
3. Teamwork	-.09*	.19***	.64***	-.08	.14**	-.07	.09
4. Learning	-.23***	-.12**	-.07	.68***	.07	.03	.09*
5. Perseverance	-.36***	-.19***	.08	.10*	.46***	.16***	.28***
6. Intrinsic motivation	-.14**	-.24***	-.05	-.01	.08	.48***	.09*
7. Goal pursuit	-.30***	-.07	.01	.19***	.28***	.13**	.43***
Frequency of human rating	0.57	0.51	0.46	0.84	0.38	0.74	0.53
Mean of computer-generated likelihood	0.30	0.24	0.20	0.42	0.21	0.39	0.25

Note. *** $p < .001$. ** $p < .01$. * $p < .05$.

Table S34. Descriptive statistics and point biserial correlations between computer-generated likelihoods and human ratings of personal qualities in the *Development Sample* for Latino applicants – admissions officers

Personal quality	1	2	3	4	5	6	7
Computer-generated likelihoods							
1. Prosocial purpose	.80***	.12***	-.13***	-.22***	-.24***	-.07	-.19***
2. Leadership	.11***	.73***	.14***	-.14***	-.08*	-.21***	.00
3. Teamwork	-.18***	.10**	.60***	-.11***	.10**	-.07*	.04
4. Learning	-.29***	-.14***	-.05	.61***	.09**	.00	.09**
5. Perseverance	-.34***	-.12***	.08*	.11**	.51***	.03	.26***
6. Intrinsic motivation	-.10**	-.30***	-.13***	.07*	.11***	.42***	.02
7. Goal pursuit	-.31***	-.10**	.14***	.16***	.29***	.02	.47***
Frequency of human rating	0.51	0.55	0.47	0.86	0.49	0.90	0.54
Mean of computer-generated likelihood	0.26	0.28	0.24	0.45	0.27	0.44	0.27

Note. *** $p < .001$. ** $p < .01$. * $p < .05$.

Table S35. Descriptive statistics and point biserial correlations between computer-generated likelihoods and human ratings of personal qualities in the *Development Sample* for Asian applicants – admissions officers

Personal quality	1	2	3	4	5	6	7
Computer-generated likelihoods							
1. Prosocial purpose	.80***	.11**	-.15***	-.27***	-.21***	-.07	-.24***
2. Leadership	.10*	.72***	.13**	-.10*	-.03	-.17***	.07
3. Teamwork	-.20***	.12**	.63***	.00	.07	-.05	.13**
4. Learning	-.34***	-.18***	-.04	.66***	.04	.02	.08
5. Perseverance	-.34***	-.11**	.12**	.11**	.52***	.08*	.26***
6. Intrinsic motivation	.00	-.22***	-.01	-.06	.09*	.44***	.10*
7. Goal pursuit	-.36***	-.01	.17***	.20***	.26***	.04	.43***
Frequency of human rating	0.61	0.43	0.43	0.98	0.41	0.76	0.42
Mean of computer-generated likelihood	0.34	0.22	0.20	0.51	0.23	0.39	0.24

Note. *** $p < .001$. ** $p < .01$. * $p < .05$.

Table S36. Descriptive statistics and point biserial correlations between computer-generated likelihoods and human ratings of personal qualities in the *Development Sample* for applicants reporting other races/ethnicities – admissions officers

Personal quality	1	2	3	4	5	6	7
Computer-generated likelihoods							
1. Prosocial purpose	.82***	.12*	-.13*	-.13*	-.21***	-.13*	-.17**
2. Leadership	.14**	.72***	.11*	-.10	-.03	-.11*	.05
3. Teamwork	-.18***	.21***	.63***	-.07	.10*	.03	.13*
4. Learning	-.21***	-.14**	-.13*	.65***	.06	-.02	.04
5. Perseverance	-.32***	-.05	.07	.03	.44***	.10*	.22***
6. Intrinsic motivation	-.11*	-.18***	-.04	-.06	.04	.49***	-.03
7. Goal pursuit	-.23***	-.02	.08	.05	.28***	.05	.46***
Frequency of human rating	0.42	0.40	0.47	0.95	0.43	0.87	0.48
Mean of computer-generated likelihood	0.24	0.22	0.23	0.48	0.26	0.45	0.26

Note. *** $p < .001$. ** $p < .01$. * $p < .05$.

Table S37. Descriptive statistics and point biserial correlations between computer-generated likelihoods and human ratings of personal qualities in the *Development Sample* for applicants who did not report their race/ethnicity – admissions officers

Personal quality	1	2	3	4	5	6	7
Computer-generated likelihoods							
1. Prosocial purpose	.73***	.13*	-.12*	-.26***	-.22***	-.07	-.16**
2. Leadership	.13*	.74***	.26***	-.17**	-.05	-.24***	-.07
3. Teamwork	-.19***	.24***	.54***	.04	.01	-.09	.14*
4. Learning	-.32***	-.10	-.01	.64***	.04	.00	.18**
5. Perseverance	-.32***	-.04	.14*	.02	.31***	.05	.26***
6. Intrinsic motivation	-.04	-.28***	-.18**	-.04	.08	.46***	-.01
7. Goal pursuit	-.30***	-.05	.14*	.14*	.19**	.04	.47***
Frequency of human rating	0.59	0.51	0.40	0.82	0.33	0.84	0.45
Mean of computer-generated likelihood	0.31	0.27	0.19	0.43	0.20	0.41	0.24

Note. *** $p < .001$. ** $p < .01$,.* $p < .05$.

Table S38. Descriptive statistics and point biserial correlations between computer-generated likelihoods and human ratings of personal qualities in the *Development Sample* for applicants with no parents with college degrees – admissions officers

Personal quality	1	2	3	4	5	6	7
Computer-generated likelihoods							
1. Prosocial purpose	.80***	.12***	-.13***	-.23***	-.21***	-.07**	-.16***
2. Leadership	.10***	.75***	.20***	-.15***	-.06*	-.13***	.00
3. Teamwork	-.17***	.22***	.63***	-.05	.08**	-.03	.07**
4. Learning	-.28***	-.15***	-.05*	.68***	.07**	.00	.12***
5. Perseverance	-.36***	-.09***	.10***	.10***	.43***	.08**	.23***
6. Intrinsic motivation	-.06*	-.23***	-.06*	-.03	.08**	.45***	.05*
7. Goal pursuit	-.30***	-.03	.09***	.18***	.23***	.07**	.41***
Frequency of human rating	0.58	0.50	0.42	0.84	0.39	0.77	0.47
Mean of computer-generated likelihood	0.31	0.25	0.20	0.44	0.22	0.39	0.24

Note. *** $p < .001$. ** $p < .01$. * $p < .05$.

Table S39. Descriptive statistics and point biserial correlations between computer-generated likelihoods and human ratings of personal qualities in the *Development Sample* for applicants with one parent with a college degree – admissions officers

Personal quality	1	2	3	4	5	6	7
Computer-generated likelihoods							
1. Prosocial purpose	.77***	.19***	-.14***	-.19***	-.26***	-.09*	-.22***
2. Leadership	.19***	.70***	.11**	-.14***	-.08	-.18***	-.05
3. Teamwork	-.20***	.06	.63***	-.04	.10**	.01	.15***
4. Learning	-.24***	-.13***	-.08*	.63***	.06	.01	.05
5. Perseverance	-.32***	-.09*	.11**	.06	.51***	.02	.29***
6. Intrinsic motivation	-.09*	-.21***	-.05	.00	.11**	.46***	.09*
7. Goal pursuit	-.28***	-.05	.18***	.11**	.32***	.01	.51***
Frequency of human rating	0.53	0.44	0.46	0.89	0.44	0.84	0.49
Mean of computer-generated likelihood	0.27	0.24	0.24	0.47	0.25	0.44	0.26

Note. *** $p < .001$. ** $p < .01$. * $p < .05$.

Table S40. Descriptive statistics and point biserial correlations between computer-generated likelihoods and human ratings of personal qualities in the *Development Sample* for applicants with two parents with college degrees – admissions officers

Personal quality	1	2	3	4	5	6	7
Computer-generated likelihoods							
1. Prosocial purpose	.84***	.12***	-.12***	-.22***	-.27***	-.10**	-.22***
2. Leadership	.12***	.73***	.14***	-.15***	-.08*	-.22***	.06
3. Teamwork	-.16***	.13***	.60***	-.15***	.11**	-.09**	.11**
4. Learning	-.31***	-.17***	-.09**	.60***	.04	.02	.06
5. Perseverance	-.35***	-.13***	.12***	.06	.55***	.13***	.28***
6. Intrinsic motivation	-.10**	-.28***	-.17***	.02	.06	.42***	-.01
7. Goal pursuit	-.35***	-.10**	.13***	.10**	.30***	.06	.46***
Frequency of human rating	0.54	0.50	0.50	0.94	0.48	0.92	0.53
Mean of computer-generated likelihood	0.28	0.26	0.24	0.49	0.26	0.45	0.27

Note. *** $p < .001$. ** $p < .01$. * $p < .05$.

Table S41. Descriptive statistics and point biserial correlations between computer-generated likelihoods and human ratings of personal qualities in the *Development Sample* for female applicants – admissions officers

Personal quality	1	2	3	4	5	6	7
Computer-generated likelihoods							
1. Prosocial purpose	.78***	.12***	-.13***	-.18***	-.23***	-.14***	-.18***
2. Leadership	.13***	.74***	.14***	-.11***	-.07**	-.15***	.02
3. Teamwork	-.17***	.15***	.62***	-.10***	.09***	-.01	.08**
4. Learning	-.25***	-.13***	-.07**	.65***	.03	.05*	.07**
5. Perseverance	-.33***	-.11***	.11***	.04	.48***	.09***	.25***
6. Intrinsic motivation	-.11***	-.24***	-.05	-.02	.09***	.46***	.06*
7. Goal pursuit	-.28***	-.05	.11***	.10***	.29***	.07**	.45***
Frequency of human rating	0.45	0.46	0.46	0.86	0.43	0.81	0.51
Mean of computer-generated likelihood	0.24	0.24	0.22	0.45	0.25	0.41	0.27

Note. *** $p < .001$. ** $p < .01$. * $p < .05$.

Table S42. Descriptive statistics and point biserial correlations between computer-generated likelihoods and human ratings of personal qualities in the *Development Sample* for male applicants – admissions officers

Personal quality	1	2	3	4	5	6	7
Computer-generated likelihoods							
1. Prosocial purpose	.81***	.14***	-.13***	-.26***	-.25***	-.06*	-.20***
2. Leadership	.12***	.73***	.18***	-.18***	-.06*	-.17***	.00
3. Teamwork	-.18***	.16***	.62***	-.05	.10***	-.06*	.12***
4. Learning	-.30***	-.17***	-.06*	.65***	.10***	-.02	.11***
5. Perseverance	-.37***	-.09***	.11***	.12***	.49***	.08***	.27***
6. Intrinsic motivation	-.06**	-.24***	-.11***	.00	.07**	.44***	.04
7. Goal pursuit	-.33***	-.05*	.13***	.19***	.25***	.05*	.44***
Frequency of human rating	0.64	0.51	0.44	0.89	0.42	0.84	0.47
Mean of computer-generated likelihood	0.34	0.26	0.21	0.46	0.23	0.43	0.24

Note. *** $p < .001$. ** $p < .01$. * $p < .05$.

Table S43. Descriptive statistics and point biserial correlations between computer-generated likelihoods and human ratings of personal qualities in the *Development Sample* for applicants with married parents – admissions officers

Personal quality	1	2	3	4	5	6	7
Computer-generated likelihoods							
1. Prosocial purpose	.82***	.13***	-.11***	-.19***	-.22***	-.08*	-.13***
2. Leadership	.16***	.73***	.21***	-.14***	-.09**	-.17***	-.02
3. Teamwork	-.18***	.19***	.67***	-.07*	.07*	-.04	.12***
4. Learning	-.26***	-.13***	-.07*	.65***	.09**	.00	.14***
5. Perseverance	-.36***	-.09**	.10**	.08*	.45***	.12***	.23***
6. Intrinsic motivation	-.09**	-.24***	-.11***	.00	.08*	.46***	.07*
7. Goal pursuit	-.28***	-.02	.11***	.14***	.24***	.08**	.40***
Frequency of human rating	0.58	0.49	0.43	0.84	0.38	0.81	0.46
Mean of computer-generated likelihood	0.29	0.24	0.21	0.45	0.23	0.41	0.24

Note. *** $p < .001$. ** $p < .01$. * $p < .05$.

Table S44. Descriptive statistics and point biserial correlations between computer-generated likelihoods and human ratings of personal qualities in the *Development Sample* for applicants with parents who are not married – admissions officers

Personal quality	1	2	3	4	5	6	7
Computer-generated likelihoods							
1. Prosocial purpose	.79***	.14***	-.15***	-.24***	-.25***	-.09***	-.22***
2. Leadership	.11***	.74***	.14***	-.15***	-.05*	-.16***	.02
3. Teamwork	-.18***	.14***	.60***	-.07***	.11***	-.03	.09***
4. Learning	-.29***	-.17***	-.07**	.65***	.05*	.02	.06**
5. Perseverance	-.34***	-.11***	.12***	.08***	.50***	.07**	.27***
6. Intrinsic motivation	-.07***	-.24***	-.07**	-.01	.08***	.44***	.03
7. Goal pursuit	-.33***	-.07**	.13***	.15***	.28***	.05*	.46***
Frequency of human rating	0.55	0.49	0.46	0.90	0.45	0.84	0.50
Mean of computer-generated likelihood	0.30	0.25	0.22	0.46	0.24	0.42	0.26

Note. *** $p < .001$. ** $p < .01$. * $p < .05$.

Table S45. Descriptive statistics and point biserial correlations between computer-generated likelihoods and human ratings of personal qualities in the *Development Sample* for English language learner applicants – admissions officers

Personal quality	1	2	3	4	5	6	7
Computer-generated likelihoods							
1. Prosocial purpose	.80***	.14***	-.13***	-.20***	-.26***	-.09***	-.19***
2. Leadership	.13***	.73***	.16***	-.13***	-.06**	-.17***	.01
3. Teamwork	-.18***	.16***	.61***	-.08***	.11***	-.03	.10***
4. Learning	-.26***	-.14***	-.06**	.64***	.08***	.02	.10***
5. Perseverance	-.35***	-.10***	.11***	.08***	.48***	.10***	.26***
6. Intrinsic motivation	-.09***	-.26***	-.09***	.01	.10***	.45***	.05*
7. Goal pursuit	-.29***	-.05*	.12***	.13***	.27***	.05**	.46***
Frequency of human rating	0.54	0.50	0.46	0.87	0.42	0.85	0.50
Mean of computer-generated likelihood	0.28	0.26	0.22	0.45	0.24	0.42	0.26

Note. *** $p < .001$. ** $p < .01$. * $p < .05$.

Table S46. Descriptive statistics and point biserial correlations between computer-generated likelihoods and human ratings of personal qualities in the *Development Sample* for native English-speaking applicants – admissions officers

Personal quality	1	2	3	4	5	6	7
Computer-generated likelihoods							
1. Prosocial purpose	.80***	.14***	-.13***	-.29***	-.19***	-.08*	-.19***
2. Leadership	.11**	.73***	.17***	-.19***	-.07*	-.16***	.01
3. Teamwork	-.17***	.16***	.64***	-.05	.05	-.05	.11**
4. Learning	-.33***	-.20***	-.08*	.67***	.02	.00	.05
5. Perseverance	-.35***	-.10**	.12***	.10**	.48***	.04	.24***
6. Intrinsic motivation	-.04	-.20***	-.07*	-.03	.04	.43***	.03
7. Goal pursuit	-.36***	-.06	.14***	.18***	.27***	.07	.38***
Frequency of human rating	0.61	0.46	0.41	0.91	0.43	0.77	0.45
Mean of computer-generated likelihood	0.33	0.23	0.19	0.47	0.22	0.40	0.24

Note. *** $p < .001$. ** $p < .01$. * $p < .05$.

Table S47. Descriptive statistics and point biserial correlations between computer-generated likelihoods and human ratings of personal qualities in the *Development Sample* for applicants who attended Title 1 public high schools – admissions officers

Personal quality	1	2	3	4	5	6	7
Computer-generated likelihoods							
1. Prosocial purpose	.79***	.13***	-.16***	-.21***	-.22***	-.07*	-.18***
2. Leadership	.16***	.79***	.17***	-.18***	-.06*	-.16***	.01
3. Teamwork	-.20***	.20***	.63***	-.08**	.08**	-.05	.12***
4. Learning	-.25***	-.20***	-.08**	.67***	.05	-.03	.09**
5. Perseverance	-.36***	-.12***	.13***	.07*	.45***	.08**	.26***
6. Intrinsic motivation	-.08**	-.28***	-.13***	-.04	.06	.47***	.06
7. Goal pursuit	-.32***	-.04	.14***	.14***	.23***	.07*	.44***
Frequency of human rating	0.56	0.52	0.47	0.86	0.41	0.82	0.49
Mean of computer-generated likelihood	0.30	0.27	0.23	0.44	0.24	0.41	0.26

Note. *** $p < .001$. ** $p < .01$. * $p < .05$.

Table S48. Descriptive statistics and point biserial correlations between computer-generated likelihoods and human ratings of personal qualities in the *Development Sample* for applicants who attended non-Title-1 high schools – admissions officers

Personal Quality	1	2	3	4	5	6	7
Computer-generated likelihoods							
1. Prosocial purpose	.81***	.14***	-.12***	-.23***	-.26***	-.09***	-.23***
2. Leadership	.11***	.70***	.13***	-.12***	-.07**	-.16***	.00
3. Teamwork	-.16***	.12***	.61***	-.06*	.12***	-.03	.09***
4. Learning	-.29***	-.12***	-.05	.63***	.08**	.03	.14***
5. Perseverance	-.36***	-.09***	.10***	.09***	.50***	.09***	.26***
6. Intrinsic motivation	-.10***	-.23***	-.05*	.01	.10***	.44***	.04
7. Goal pursuit	-.32***	-.08**	.11***	.15***	.28***	.04	.47***
Frequency of human rating	0.56	0.49	0.46	0.88	0.45	0.84	0.51
Mean of computer-generated likelihood	0.29	0.25	0.23	0.47	0.25	0.43	0.26

Note. *** $p < .001$. ** $p < .01$. * $p < .05$.

Section 6. Quality Check of Imputation Procedure for Missing Data

Figure S2 shows distributions of each of the variables with missing data. We show the original distributions in black, and the overlapping red distributions represent the $m = 25$ imputed datasets. As shown in **Figure S2,** imputed distributions closely resemble the original distribution, suggesting adequate imputation quality.

Figure S2. Imputation quality. The $m = 25$ imputed datasets closely matched the existing data, suggesting adequate imputation quality.

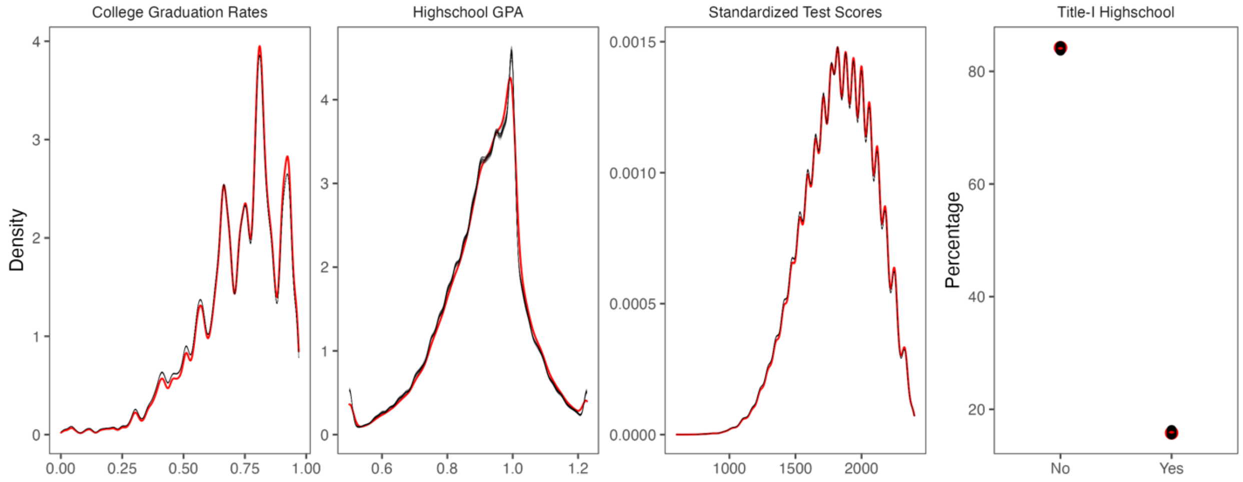

Section 7. Details on Model Interpretation

As shown in **Figure S3,** word attribution scores for RoBERTa models trained on admissions officers' ratings are face valid and roughly correspond with word attribution scores for models trained on research assistants' ratings.

Figure S3. Complete or partial words on which RoBERTa models finetuned on admissions officers relied most for generating personal quality scores. Font size is proportional to word importance. Darker words are more common. Words importance is not invariant across essays, it depends on word context. Word importance and frequency were largely independent (r = -.03, p < .001). For instance, for intrinsic motivation, the model relied more on the word "pleasure" then the word "fun," but essays were more likely to contain the word "fun" then the word "pleasure."

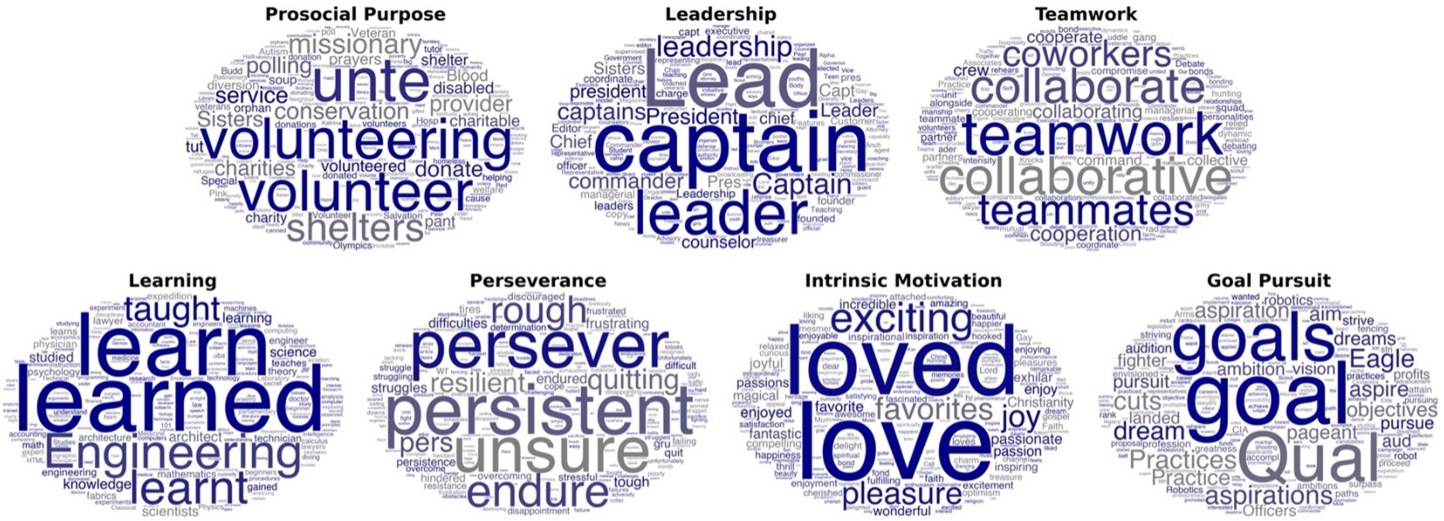

Section 8. Details on Models Predicting Graduation Directly from Text

To estimate a ceiling on how much language could be predictive of graduation, we fine-tuned a model where student writing was used to predict whether students graduated. To reduce computational load, we used a random sample of 18,000 students. We used a random subset of 70% of this subset for training, and the remaining 30% was split in half for validation and testing. The out-of-sample AUC of the model was .626. We compared this to a model trained on the same subsets of data, where we trained a logistic regression model to predict college graduation from our 7 computer-generated likelihoods of personal qualities. This model obtained an out-of-sample AUC of .557 and .568, for research assistants and admissions officers, respectively. We suggest that the difference in these AUCs (ΔAUC = .069 and .058, for research assistants and admissions officers, respectively) suggests the existence of variance in students writing that is predictive of college graduation which is *not* encoded on our measures of personal qualities. These might capture variance related to demographic characteristics, gender, or cognitive ability. The existence of such variance might help make sense of our findings in light of other studies (e.g., Alvero et al., (*18*)) which suggest that student writing *does* encode demographic characteristics.

Figure S4 shows the word tokens with the highest positive and negative attribution scores, that is the words and fractions of words that the model tended to use to classify students. Interestingly, misspellings (e.g., “alot”), exclamation marks (“!”), and informal language (e.g., “guys”) tend to receive negative attribution scores.

Figure S4. Attribution scores for word tokens in a model where student writing directly predicts graduation. Font size is proportional to word importance. Darker words are more common. Words importance is not invariant across essays, it depends on word context. Both words clouds come from the same model, but they are words associated with negative (left) and positive (right) predictions of graduation.

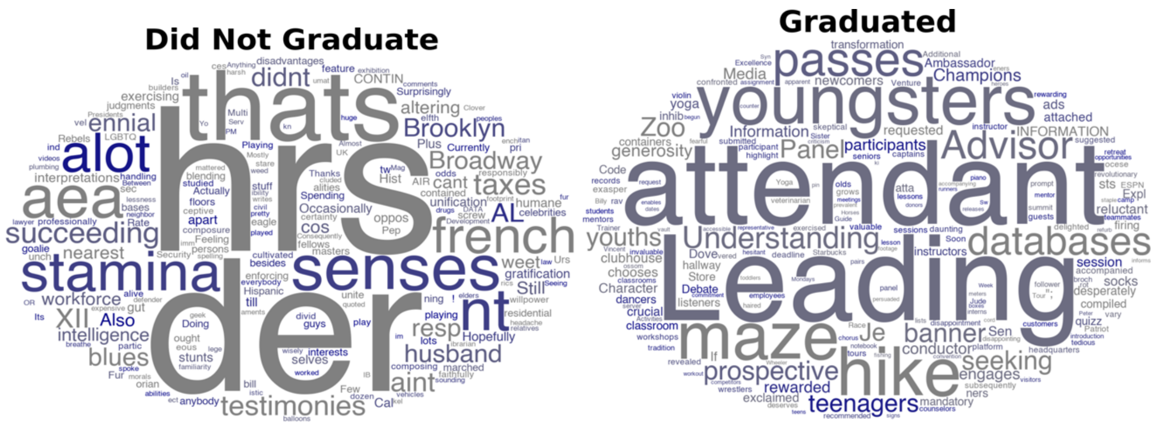

To aid the interpretation of these models, we used Linguistic Inquiry and Word Count (LIWC-22 (48)) to identify linguistic correlates of model predictions. We calculated bivariate correlations between each LIWC variable, and the model’s predicted probability of graduation based solely on student writing. As shown in **Figure S5**, after correcting for multiple comparisons, it seems that the model finds indications of educational/cultural level (e.g., word count, words with more than 6 letters, punctuation, Analytic language); but also words related to personal qualities (e.g., prosocial, social words, and moral words).

Figure S5. Correlations between LIWC variables and predicted probability of graduation. Values are Pearson correlation coefficients. They have been Bonferroni-corrected for multiple comparisons. Only the 59 surviving significant correlations out of a potential 117 are shown. Refer to the LIWC Manual (48) for definitions and example words of LIWC variables.

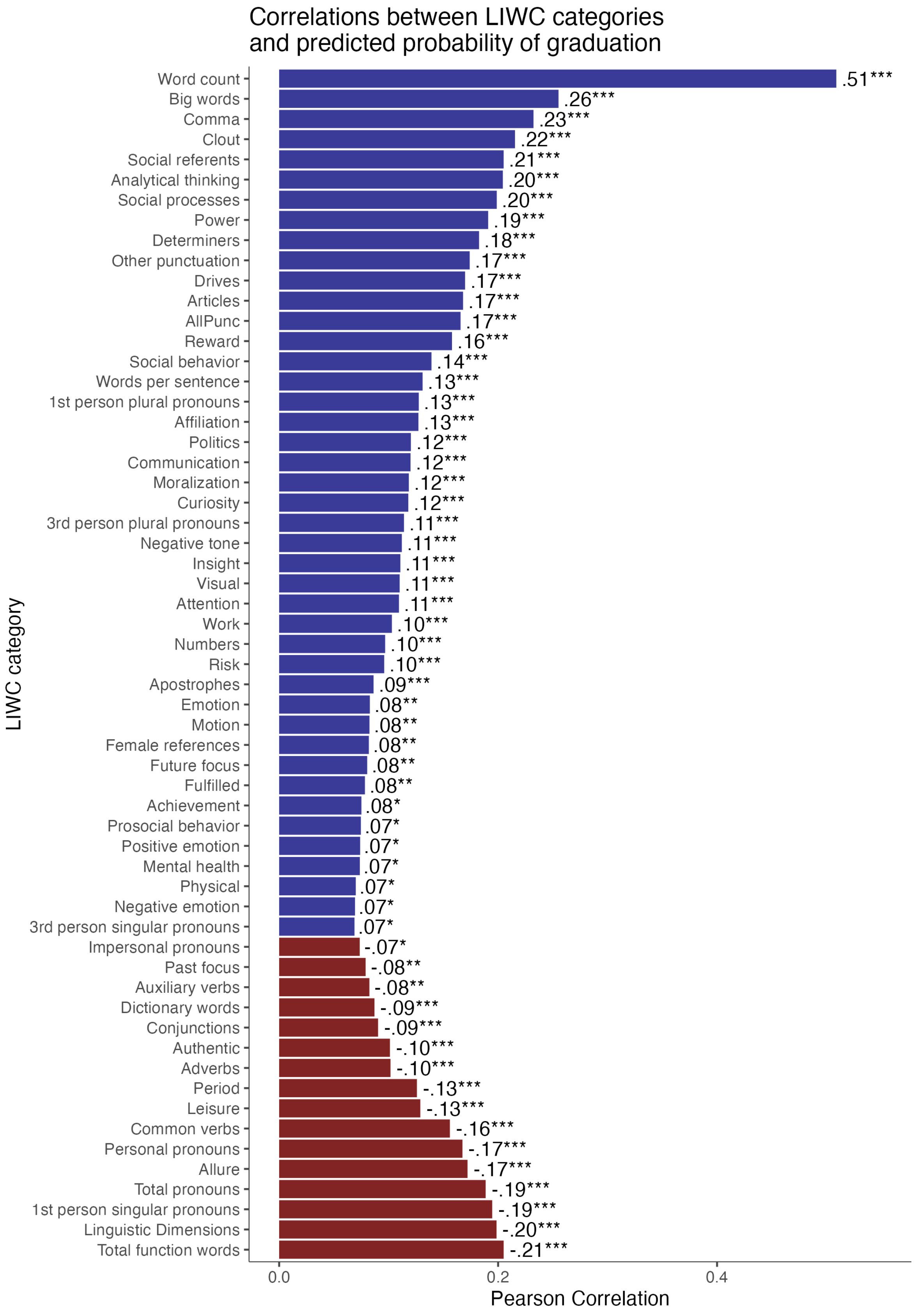

Section 9. Details on Interaction Effects of Demographics with Personal Qualities Predicting Graduation

We tested the equality of predictive validity of computer-generated likelihoods of personal qualities by fitting model (2) in **Table 2** in the main text but including interaction terms between each personal quality and standardized test scores and each demographic characteristic. **Figures S6** and **S7** below shows the coefficients for each interaction term with statistical significance denoted with asterisks. These coefficients should be interpreted as the difference between the coefficient for the reference class, and the specified demographic category. For example, the coefficient in the top left of **Figure S6** indicates that prosocial purpose is .02 less predictive for English language learners as opposed to native speakers, the lack of asterisks means that the difference is not significant. As shown in **Figures S6** and **S7,** computer-generated likelihoods of personal qualities were equally predictive of college graduation across demographics, suggesting fairness across subgroups in terms of predictive validity.

Further, we investigated whether *intersections* of two demographic characteristics might be associated with higher or lower predictive accuracy. To test this, we conducted subgroup analyses in which we calculated the predictive accuracy of personal qualities for every possible intersection of two characteristics (e.g., Black English language learners, women in Title-I high schools, etc.). We then used a Wald test against the null hypothesis that all coefficients were equal. We used multiple-comparison corrected z-tests to then identify significant differences if the null hypothesis was rejected. We found no consistent or theoretically interpretable pattern in these intersectional analyses. As shown in **Table S47** and **S48**, 12 out of potentially 45,087 comparisons (< 0.03%) were significantly different, for models trained on research assistants. There were no differences for models trained on admissions officers.

Figure S6

Coefficients of interaction terms between personal qualities and demographic characteristics in the prediction of six-year college graduation – research assistant model.

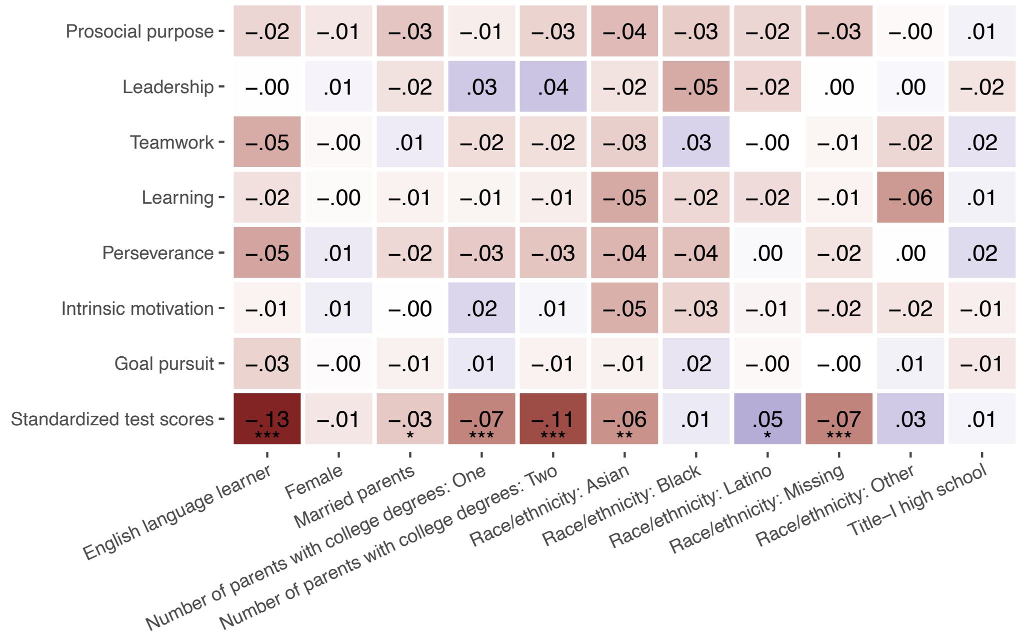

Figure S7

Coefficients of interaction terms between personal qualities and demographic characteristics in the prediction of six-year college graduation – admissions officer model.

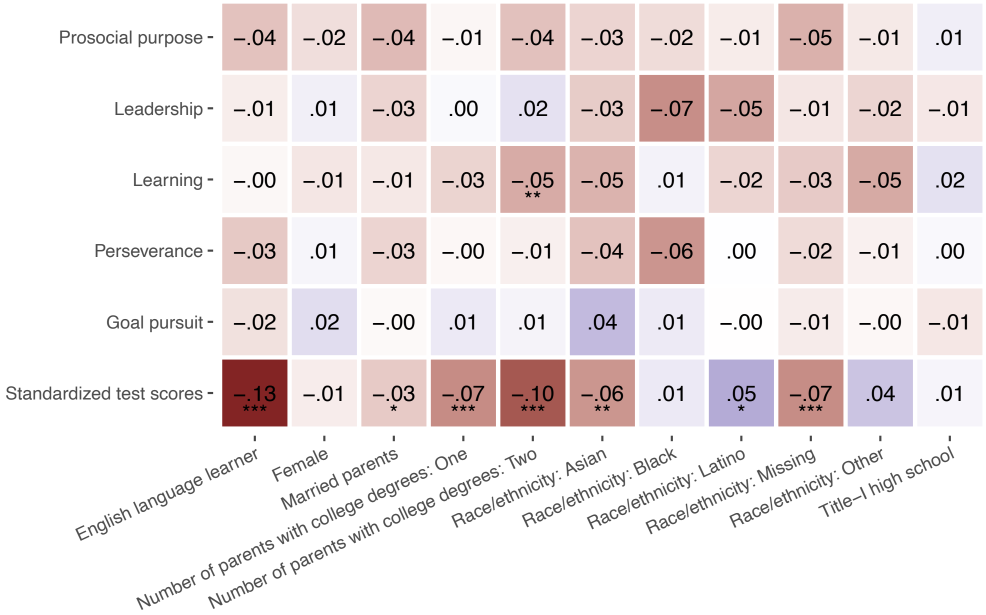

Table S49. Wald tests for equality of coefficients of personal qualities on college graduation across intersections of demographic characteristics.

Term	Research Assistants			Admissions Officers		
	χ^2	*p*	*Post-hoc differences*	χ^2	*p*	*Post-hoc differences*
Prosocial Purpose	170.124	.003	1 out of 6,441	187.357	< .001	-
Leadership	170.225	.003	-	248.421	< .001	-
Teamwork	143.153	.204	-	221.471	< .001	-
Learning	175.138	.001	-	157.869	.024	-
Perseverance	205.324	< .001	11 out of 6,441	120.947	> .999	-
Intrinsic motivation	112.226	> .999	-	95.373	> .999	-
Goal pursuit	72.868	> .999	-	107.387	> .999	-

Note. Overall, 0.03% of all possible differences were significant for research assistants, and no differences (0.00%) were significant for admissions officers. *p*-values are Bonferroni corrected for multiple comparisons.

Table S50. Pairwise differences of coefficients of personal qualities on college graduation across intersections of demographic characteristics.

	Reference		Comparison		Difference	*Z*	*p*
	B	SE	B	SE			
**Prosocial was more predictive of graduation for students with married parents of other race/ethnicity than…**							
English language learners with two parents with college degrees	0.37	0.07	−0.03	0.05	0.39	4.59	0.03
**Perseverance was less predictive of graduation for students with two parents with college degrees that are English language learners than…**							
Females in Title-I high schools	−0.24	0.06	0.12	0.05	−0.36	−4.60	0.03
Students whose parents are unmarried and have no college degrees	−0.24	0.06	0.13	0.05	−0.37	−4.78	0.01
Females whose parents are not married	−0.24	0.06	0.10	0.04	−0.35	−4.54	0.04
Whites whose parents have no college degrees	−0.24	0.06	0.10	0.04	−0.34	−4.53	0.04
Native speakers in Title-I high schools	−0.24	0.06	0.09	0.04	−0.33	−4.52	0.04
Females whose parents have no college degrees	−0.24	0.06	0.11	0.04	−0.35	−4.90	0.01
Students in Title-I high schools whose parents are not married	−0.24	0.06	0.08	0.03	−0.32	−4.57	0.03
Native speakers whose parents are not married	−0.24	0.06	0.08	0.03	−0.33	−4.71	0.02
Native speakers whose parents have no college degrees	−0.24	0.06	0.08	0.03	−0.32	−4.61	0.03
Female native speakers	−0.24	0.06	0.06	0.02	−0.30	−4.59	0.03
White native speakers	−0.24	0.06	0.05	0.02	−0.29	−4.48	0.05

Note. Only significant pairwise differences shown. *p*-values are Bonferroni corrected for multiple comparisons.

Section 10. Robustness Checks for Analyses Predicting College Graduation

Predictive Validity of Human Ratings of Personal Qualities in The Development Sample

As shown in **Table S51,** in binary logistic regression models predicting college graduation, coefficients for human ratings of personal qualities in the *Development Sample* were similar to those of computer-generated likelihoods in the *Holdout Sample.*

Predictive Validity of Computer-Generated Likelihoods of Personal Qualities Controlling for High School GPA in The Holdout Sample

In the year of data collection, high school counselors had the option to submit report card grades either online or by uploading hard-copy transcripts. Because hard-copy transcripts were not possible to de-identify, we had access to only a subset of n = 43,597 applications in the holdout sample with high school grade point average (HSGPA). **Table S52** shows results of our main model specification including HSGPA as a predictor in that subsample.

Predictive Validity of Computer-Generated Likelihoods of Personal Qualities Controlling for Institutional Graduation Rates in The Holdout Sample

As shown in **Table S53,** in binary logistic regression models predicting college graduation, coefficients for computer-generated likelihoods of personal qualities were similar in magnitude to those presented in the main text.

Table S51. Binary logistic regression models predicting college graduation from human ratings of personal qualities in the *Development Sample*

	Research Assistants		Admissions Officers	
	(1)	(2)	(1)	(2)
Human ratings of personal qualities				
Prosocial purpose	1.063	1.059	1.185***	1.131*
	(0.041)	(0.052)	(0.049)	(0.057)
Leadership	1.174***	1.066	1.165***	1.038
	(0.048)	(0.052)	(0.048)	(0.051)
Teamwork	1.011	0.954	1.081	1.019
	(0.040)	(0.045)	(0.043)	(0.049)
Mastery orientation	1.137***	1.126*	1.134**	1.055
	(0.044)	(0.054)	(0.045)	(0.052)
Perseverance	1.048	0.988	1.111**	1.042
	(0.041)	(0.047)	(0.045)	(0.051)
Intrinsic motivation	1.037	1.055	1.194***	1.126*
	(0.040)	(0.050)	(0.047)	(0.054)
Goal pursuit	1.051	1.011	1.027	0.997
	(0.041)	(0.048)	(0.041)	(0.048)
Race/ethnicity (vs. White)				
Black		1.150		1.141
		(0.180)		(0.177)
Latino		0.776		0.785
		(0.126)		(0.126)
Asian		1.000		1.051
		(0.173)		(0.182)
Other		0.954		0.959
		(0.166)		(0.165)
No race reported		0.789		0.797
		(0.127)		(0.127)
Parental education (vs. no parent w/ college degree)				
One parent w/ college degree		1.282		1.282*
		(0.163)		(0.162)
Two parents w/ college degree		1.544**		1.554**
		(0.210)		(0.210)
Female		1.482***		1.486***
		(0.148)		(0.146)
Married parents		1.138		1.137
		(0.117)		(0.116)
English language learner		1.164		1.110
		(0.160)		(0.151)
Title 1 high school		1.181		1.185
		(0.121)		(0.120)
Out-of-school activities (OSA)				
Number of OSA		1.199**		1.182**
		(0.066)		(0.065)
Time per OSA		1.079		1.076
		(0.058)		(0.058)
Proportion sports		1.111*		1.115*
		(0.056)		(0.056)
Standardized test scores		1.866***		1.842***
		(0.116)		(0.114)
Constant	1.975***	1.459**	1.998***	1.460**
	(0.076)	(0.208)	(0.077)	(0.206)
AUC	.565	.719	.587	.720
N	3,078	2,484	3,131	2,529

Note. *** $p < .001$. ** $p < .01$. * $p < .05$.

Table S52. Binary logistic regression models predicting college graduation from computer-generated likelihoods of personal qualities controlling for high school GPA in the *Holdout Sample*

	Research Assistants		Admissions Officers	
	(1)	(2)	(1)	(2)
Human ratings of personal qualities				
Prosocial purpose	1.113***	1.068***	1.225***	1.101***
	(0.081)	(0.090)	(0.096)	(0.109)
Leadership	1.132***	1.063***	1.207***	1.080***
	(0.081)	(0.090)	(0.090)	(0.099)
Teamwork	1.040**	0.993	1.088***	1.019
	(0.079)	(0.089)	(0.083)	(0.094)
Mastery orientation	1.054***	1.038**	1.109***	1.009
	(0.078)	(0.085)	(0.084)	(0.097)
Perseverance	1.072***	1.019	1.097***	1.051**
	(0.085)	(0.088)	(0.094)	(0.104)
Intrinsic motivation	1.063***	1.011	1.145***	1.023
	(0.077)	(0.085)	(0.082)	(0.094)
Goal pursuit	1.046***	1.019	1.046**	1.023
	(0.078)	(0.087)	(0.093)	(0.099)
Race/ethnicity (vs. White)				
Black		0.819***		0.818***
		(0.370)		(0.349)
Latino		0.935		0.932
		(0.343)		(0.336)
Asian		0.760***		0.762***
		(0.319)		(0.324)
Other		0.758***		0.756***
		(0.299)		(0.273)
No race reported		0.839***		0.842***
		(0.233)		(0.262)
Parental education (vs. no parent w/ college degree)				
One parent w/ college degree		1.261***		1.261***
		(0.224)		(0.228)
Two parents w/ college degree		1.455***		1.455***
		(0.220)		(0.223)
Female		1.385***		1.379***
		(0.180)		(0.176)
Married parents		1.339***		1.335***
		(0.200)		(0.198)
English language learner		0.711***		0.714***
		(0.291)		(0.293)
Title 1 high school		0.909**		0.909**
		(0.218)		(0.219)
Out-of-school activities				
Number of OSA		1.203***		1.197***
		(0.088)		(0.085)
Time per OSA		1.081***		1.077***
		(0.081)		(0.081)
Proportion sports		1.063***		1.057***
		(0.085)		(0.088)
Standardized test scores		1.242***		1.239***
		(0.104)		(0.103)
HSGPA		1.441***		1.437***
		(0.096)		(0.094)
Constant	3.480***	2.446***	3.503***	2.455***
	(0.077)	(0.266)	(0.077)	(0.259)
AUC	.554	.702	.569	.703
N	43,591	43,591	43,591	43,591

Note. *** $p < .001$. ** $p < .01$. * $p < .05$.

Table S53. Binary logistic regression models predicting college graduation controlling for institutional graduation rates from human ratings of personal qualities in the *Holdout Sample*

	Research Assistants		Admissions Officers	
	(1)	(2)	(1)	(2)
Human ratings of personal qualities				
Prosocial purpose	1.132***	1.063***	1.252***	1.093***
	(0.080)	(0.090)	(0.101)	(0.111)
Leadership	1.133***	1.055***	1.214***	1.065***
	(0.082)	(0.096)	(0.087)	(0.101)
Teamwork	1.080***	1.023***	1.135***	1.052***
	(0.083)	(0.093)	(0.086)	(0.091)
Mastery orientation	1.065***	1.036***	1.146***	1.018***
	(0.079)	(0.087)	(0.086)	(0.096)
Perseverance	1.071***	1.000	1.089***	1.036***
	(0.082)	(0.089)	(0.093)	(0.104)
Intrinsic motivation	1.068***	1.005	1.142***	0.996
	(0.078)	(0.087)	(0.083)	(0.094)
Goal pursuit	1.041***	0.995	1.048***	1.026***
	(0.080)	(0.085)	(0.095)	(0.098)
Race/ethnicity (vs. White)				
Black		0.754***		0.755***
		(0.328)		(0.338)
Latino		0.857***		0.857***
		(0.306)		(0.361)
Asian		0.696***		0.700***
		(0.350)		(0.314)
Other		0.733***		0.735***
		(0.305)		(0.320)
No race reported		0.828***		0.832***
		(0.244)		(0.244)
Parental education (vs. no parent w/ college degree)				
One parent w/ college degree		1.156***		1.157***
		(0.231)		(0.230)
Two parents w/ college degree		1.196***		1.199***
		(0.234)		(0.219)
Female		1.465***		1.463***
		(0.183)		(0.177)
Married parents		1.281***		1.277***
		(0.196)		(0.202)
English language learner		0.684***		0.688***
		(0.290)		(0.289)
Title 1 high school		0.974*		0.970**
		(0.230)		(0.251)
Institutional graduation rates		1.891***		1.880***
		(0.095)		(0.092)
Out-of-school activities				
Number of OSA		1.159***		1.152***
		(0.089)		(0.091)
Time per OSA		1.082***		1.079***
		(0.082)		(0.083)
Proportion sports		1.029***		1.021***
		(0.084)		(0.086)
Standardized test scores		1.164***		1.162***
		(0.107)		(0.104)
Constant	3.558***	2.986***		2.985***
	(0.004)	(0.277)		(0.260)
AUC	.560	.741	.576	.741
N	306,463	306,463	306,463	306,463

Note. *** $p < .001$. ** $p < .01$. * $p < .05$.

REFERENCES AND NOTES

1. National Research Council, *Assessing 21st Century Skills: Summary of a Workshop* (The National Academies Press, 2011).

2. T. E. Moffitt, L. Arseneault, D. Belsky, N. Dickson, R. J. Hancox, H. Harrington, R. Houts, R. Poulton, B. W. Roberts, S. Ross, M. R. Sears, W. M. Thomson, A. Caspi, A gradient of childhood self-control predicts health, wealth, and public safety. *Proc. Natl. Acad. Sci.* **108**, 2693–2698 (2011).

3. M. Almlund, A. L. Duckworth, J. Heckman, T. Kautz, Personality Psychology and Economics. *Handbook of the Economics of Education* (Elsevier, 2011), vol. 4, pp. 1–181.

4. S. B. Robbins, K. Lauver, H. Le, D. Davis, R. Langley, A. Carlstrom, Do psychosocial and study skill factors predict college outcomes? A meta-analysis *Psychol. Bull.* **130**, 261–288 (2004).

5. P. C. Kyllonen, A. A. Lipnevich, J. Burrus, R. D. Roberts, Personality, motivation, and college readiness: A prospectus for assessment and development. *ETS Res. Rep. Ser.* **2014**, 1–48 (2014).

6. A. L. Coleman, J. L. Keith, Understanding Holistic Review in Higher Education Admissions, *Tech. rep.*, College Board (2018).

7. J. Karabel, *The Chosen: The Hidden History of Admission and Exclusion at Harvard, Yale, and Princeton.* (Houghton Mifflin Harcourt, 2005).

8. M. N. Bastedo, N. A. Bowman, K. M. Glasener, J. L. Kelly, What are we talking about when we talk about holistic review? Selective college admissions and its effects on low-SES students. *J. High. Educ.* **89**, 782–805 (2018).

9. R. Starkman, Confessions of an Application Reader. *The New York Times* (2013).

10. T. R. Anderson, R. Weissbourd, Character Assessment in College Admission, *Tech. rep.*, Making Caring Common Project (2020).

11. D. Kahneman, O. Sibony, C. R. Sunstein, *Noise: A Flaw in Human Judgment* (Harper Collins, 2021).

12. J. P. Rushton, C. J. Brainerd, M. Pressley, Behavioral development and construct validity: The principle of aggregation. *Psychol. Bull.* **94**, 18 (1983), 38.

13. E. Hoover, Working smarter, not harder, in admissions. *The Chronicle of Higher Education* (2017).

14. M. Korn, Some elite colleges review an application in 8 minutes (or less). *Wall Street Journal* (2018).

15. L. Tay, S. E. Woo, L. Hickman, B. M. Booth, S. D'Mello, A conceptual framework for investigating and mitigating machine-learning measurement bias (MLMB) in psychological assessment. *Adv. Methods Pract. Psychol. Sci.* **5**, 1–30 (2022).

16. L. Hickman, N. Bosch, V. Ng, R. Saef, L. Tay, S. E. Woo, Automated video interview personality assessments: Reliability, validity, and generalizability investigations. *J. Appl. Psychol.*, **107**, 1323–1351 2022.

17. J. Manyika, J. Silberg, B. Presten, What do we do about the biases in AI? *Harvard Business Review* pp. 1–5 (2019).

18. Z. Obermeyer, B. Powers, C. Vogeli, S. Mullainathan, Dissecting racial bias in an algorithm used to manage the health of populations. *Science* **366**, 447–453 (2019).

19. D. Ensign, S. A. Friedler, S. Neville, C. Scheidegger, S. Venkatasubramanian, Runaway feedback loops in predictive policing. *Proc. Mach. Learn. Res.* **81**, 160–171 (2018).

20. A. Alvero, S. Giebel, B. Gebre-Medhin, A. L. Antonio, M. L. Stevens, B. W. Domingue, Essay content and style are strongly related to household income and SAT scores: Evidence from 60,000 undergraduate applications. *Sci. Adv.* **7**, eabi9031 (2021).

21. M. O. Riedl, Human-centered artificial intelligence and machine learning. *Hum. Behav. Emerg. Technol.* **1**, 33–36 (2019).

22. B. Shneiderman, Human-centered artificial intelligence: Three fresh ideas. *AIS Trans. Hum.-Comput. Interact.* **12**, 109–124 (2020).

23. D. Gunning, M. Stefik, J. Choi, T. Miller, S. Stumpf, G.-Z. Yang, XAI–Explainable artificial intelligence. *Sci. Robot.* **4**, eaay7120 (2019).

24. Our Selection Process (2021).

25. O. Lobel, *The Equality Machine: Harnessing Digital Technology for a Brighter, More Inclusive Future* (Public Affairs, 2022).

26. Y. Liu, M. Ott, N. Goyal, J. Du, M. Joshi, D. Chen, O. Levy, M. Lewis, L. Zettlemoyer, V. Stoyanov, RoBERTa: A Robustly Optimized BERT Pretraining Approach (2019). ArXiv:1907.11692 [cs].

27. C. Pierse, Transformers Interpret (2021). Original-date: 2020–05-27T20:32:08Z.

28. J. D. Janizek, P. Sturmfels, S.-I. Lee, Explaining Explanations: Axiomatic Feature Interactions for Deep Networks (2020).

29. Y. Benjamini, D. Yekutieli, The control of the false discovery rate in multiple testing under dependency. *Ann. Statist.* **29**, 1165–1188 (2001).

30. L. Kamas, A. Preston, Empathy, gender, and prosocial behavior. *J. Behav. Exp. Econ.* **92**, 101654 (2021).

31. J. W. Pennebaker, C. K. Chung, J. Frazee, G. M. Lavergne, D. I. Beaver, When small words foretell academic success: The case of college admissions essays. *PLOS ONE* **9**, e115844 (2014).

32. J. P. Goyer, G. M. Walton, D. S. Yeager, The role of psychological factors and institutional channels in predicting the attainment of postsecondary goals. *Dev. Psychol.* **57**, 73–86 (2021).

33. M. J. Salganik, I. Lundberg, A. T. Kindel, C. E. Ahearn, K. Al-Ghoneim, A. Almaatouq, D. M. Altschul, J. E. Brand, N. B. Carnegie, R. J. Compton, D. Datta, T. Davidson, A. Filippova, C. Gilroy, B. J. Goode, E. Jahani, R. Kashyap, A. Kirchner, S. McKay, A. C. Morgan, A. Pentland, K. Polimis, L. Raes, D. E. Rigobon, C. V. Roberts, D. M. Stanescu, Y. Suhara, A. Usmani, E. H. Wang, M. Adem, A. Alhajri, B. AlShebli, R. Amin, R. B. Amos, L. P. Argyle, L. Baer-Bositis, M. Büchi, B.-R. Chung, W. Eggert, G. Faletto, Z. Fan, J. Freese, T. Gadgil, J. Gagné, Y. Gao, A. Halpern-Manners, S. P. Hashim, S. Hausen, G. He, K. Higuera, B. Hogan, I. M. Horwitz, L. M. Hummel, N. Jain, K. Jin, D. Jurgens, P. Kaminski, A. Karapetyan, E. H. Kim, B. Leizman, N. Liu, M. Möser, A. E. Mack, M. Mahajan, N. Mandell, H. Marahrens, D. Mercado-Garcia, V. Mocz, K. Mueller-Gastell, A. Musse, Q. Niu, W. Nowak, H. Omidvar, A. Or, K. Ouyang, K. M. Pinto, E. Porter, K. E. Porter, C. Qian, T. Rauf, A. Sargsyan, T. Schaffner, L. Schnabel, B. Schonfeld, B. Sender, J. D. Tang, E. Tsurkov, A. van Loon, O. Varol, X. Wang, Z. Wang, J. Wang, F. Wang, S. Weissman, K. Whitaker, M. K. Wolters, W. L. Woon, J. Wu, C. Wu, K. Yang, J. Yin, B. Zhao, C. Zhu, J. Brooks-Gunn, B. E. Engelhardt, M. Hardt, D. Knox, K. Levy, A. Narayanan, B. M. Stewart, D. J. Watts, S. McLanahan, Measuring the predictability of life outcomes with a scientific mass collaboration. *Proc. Natl. Acad. Sci.* **117**, 8398–8403 (2020).

34. T. Martin, J. M. Hofman, A. Sharma, A. Anderson, D. J. Watts, *Proceedings of the 25th International Conference on World Wide Web* (International World Wide Web Conferences Steering Committee, Montréal Québec Canada, 2016), pp. 683–694.

35. S. Ahadi, E. Diener, Multiple determinants and effect size. *J. Pers. Soc. Psychol.* **56**, 398–406 (1989).

36. F. M. Götz, S. D. Gosling, P. J. Rentfrow, Small effects: The indispensable foundation for a cumulative psychological science. *Perspect. Psychol. Sci.* **17**, 205–215 (2022).

37. S. Goldrick-Rab, Following their every move: An investigation of social-class differences in college pathways. *Sociol. Educ.* **79**, 61–79 (2006).

38. D. Hepworth, B. Littlepage, K. Hancock, Factors influencing university student academic success. *Educ. Res. Q.* **42**, 45–61 (2018).

39. S. F. Porchea, J. Allen, S. Robbins, R. P. Phelps, Predictors of long-term enrollment and degree outcomes for community college students: Integrating academic, psychosocial, socio-demographic, and situational factors. *J. High. Educ.* **81**, 680–708 (2010).

40. M. C. Murphy, M. Gopalan, E. R. Carter, K. T. U. Emerson, B. L. Bottoms, G. M. Walton, A customized belonging intervention improves retention of socially disadvantaged students at a broad-access university. *Sci. Adv.* **6**, eaba4677 (2020).

41. W. W. Willingham, *Success in College: The Role of Personal Qualities and Academic Ability* (College Board Publications, 1985).

42. J. D. W. Clifton, Managing validity versus reliability trade-offs in scale-building decisions. *Psychol. Methods* **25**, 259–270 2020.

43. D. T. Campbell, Assessing the impact of planned social change. *Eval. Program Plann.* **2**, 67–90 (1979).

44. B. J. Dietvorst, J. P. Simmons, C. Massey, Algorithm aversion: People erroneously avoid algorithms after seeing them err. *J. Exp. Psychol. Gen.* **144**, 114–126 (2015).

45. J. L. Mahoney, R. W. Larson, J. S. Eccles, H. Lord, Organized activities as developmental contexts for children and adolescents. *Organized activities as contexts of development: Extracurricular activities, after-school and community programs* pp. 3–22 (2005).

46. R. W. Larson, Toward a psychology of positive youth development. *Am. Psychol.* **55**, 170–183 2000.

47. ACT, ACT-SAT concordance: A tool for comparing scores (2013).

48. S. van Buuren, K. Groothuis-Oudshoorn, mice: Multivariate imputation by chained equations in R. *J. Stat. Softw.* **45** 1–67 (2011).

49. S. Hutt, M. Gardener, D. Kamentz, A. L. Duckworth, S. K. D'Mello, *Proceedings of the 8th International Conference on Learning Analytics and Knowledge* (ACM, Sydney New South Wales Australia, 2018), pp. 280–289.

50. R. Boyd, A. Ashokkumar, S. Seraj, J. Pennebaker, *The Development and Psychometric Properties of LIWC-22* (2022).
